# ESRP1 controls biogenesis and function of a large abundant multiexon circRNA

**DOI:** 10.1093/nar/gkad1138

**Published:** 2023-11-28

**Authors:** Dawei Liu, B Kate Dredge, Andrew G Bert, Katherine A Pillman, John Toubia, Wenting Guo, Boris J A Dyakov, Melodie M Migault, Vanessa M Conn, Simon J Conn, Philip A Gregory, Anne-Claude Gingras, Dinshaw Patel, Baixing Wu, Gregory J Goodall

**Affiliations:** Centre for Cancer Biology, SA Pathology and University of South Australia, Adelaide, SA 5000, Australia; Centre for Cancer Biology, SA Pathology and University of South Australia, Adelaide, SA 5000, Australia; Centre for Cancer Biology, SA Pathology and University of South Australia, Adelaide, SA 5000, Australia; Centre for Cancer Biology, SA Pathology and University of South Australia, Adelaide, SA 5000, Australia; School of Molecular and Biomedical Science, University of Adelaide, Adelaide, SA 5005, Australia; Centre for Cancer Biology, SA Pathology and University of South Australia, Adelaide, SA 5000, Australia; ACRF Cancer Genomics Facility, Centre for Cancer Biology, SA Pathology and University of South Australia, Frome Road, Adelaide, SA 5000, Australia; Guangdong Provincial Key Laboratory of Malignant Tumor Epigenetics and Gene Regulation, Guangdong-Hong Kong Joint Laboratory for RNA Medicine, RNA Biomedical Institute, Medical Research Center, Sun Yat-Sen Memorial Hospital, Sun Yat-Sen University, Guangzhou, 510120, China; Lunenfeld-Tanenbaum Research Institute, Mount Sinai Hospital, Sinai Health, 600 University Ave, Toronto, ON M5G 1X5, Canada; Department of Molecular Genetics, University of Toronto, Toronto, ON M5S 1A8, Canada; Centre for Cancer Biology, SA Pathology and University of South Australia, Adelaide, SA 5000, Australia; Centre for Cancer Biology, SA Pathology and University of South Australia, Adelaide, SA 5000, Australia; Flinders Health and Medical Research Institute, College of Medicine & Public Health, Flinders University, Bedford Park, SA, 5042, Australia; Centre for Cancer Biology, SA Pathology and University of South Australia, Adelaide, SA 5000, Australia; Flinders Health and Medical Research Institute, College of Medicine & Public Health, Flinders University, Bedford Park, SA, 5042, Australia; Centre for Cancer Biology, SA Pathology and University of South Australia, Adelaide, SA 5000, Australia; Lunenfeld-Tanenbaum Research Institute, Mount Sinai Hospital, Sinai Health, 600 University Ave, Toronto, ON M5G 1X5, Canada; Department of Molecular Genetics, University of Toronto, Toronto, ON M5S 1A8, Canada; Structural Biology Program, Memorial Sloan Kettering Cancer Center, New York, NY, USA; Guangdong Provincial Key Laboratory of Malignant Tumor Epigenetics and Gene Regulation, Guangdong-Hong Kong Joint Laboratory for RNA Medicine, RNA Biomedical Institute, Medical Research Center, Sun Yat-Sen Memorial Hospital, Sun Yat-Sen University, Guangzhou, 510120, China; Centre for Cancer Biology, SA Pathology and University of South Australia, Adelaide, SA 5000, Australia; School of Molecular and Biomedical Science, University of Adelaide, Adelaide, SA 5005, Australia; Adelaide Medical School, Faculty of Health and Medical Sciences, University of Adelaide, Adelaide, SA, Australia

## Abstract

While the majority of circRNAs are formed from infrequent back-splicing of exons from protein coding genes, some can be produced at quite high level and in a regulated manner. We describe the regulation, biogenesis and function of circDOCK1(2–27), a large, abundant circular RNA that is highly regulated during epithelial-mesenchymal transition (EMT) and whose formation depends on the epithelial splicing regulator ESRP1. CircDOCK1(2–27) synthesis in epithelial cells represses cell motility both by diverting transcripts from DOCK1 mRNA production to circRNA formation and by direct inhibition of migration by the circRNA. HITS-CLIP analysis and CRISPR-mediated deletions indicate ESRP1 controls circDOCK1(2–27) biosynthesis by binding a GGU-containing repeat region in intron 1 and detaining its splicing until Pol II completes its 157 kb journey to exon 27. Proximity-dependent biotinylation (BioID) assay suggests ESRP1 may modify the RNP landscape of intron 1 in a way that disfavours communication of exon 1 with exon 2, rather than physically bridging exon 2 to exon 27. The X-ray crystal structure of RNA-bound ESRP1 qRRM2 domain reveals it binds to GGU motifs, with the guanines embedded in clamp-like aromatic pockets in the protein.

## Graphical Abstract

**Graphical Abstract:**
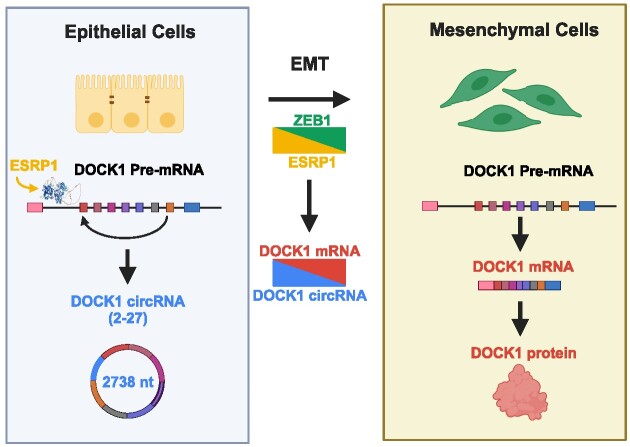

## Introduction

It is now well established that eukaryotic cells express many circular RNAs (circRNAs). Some circRNAs are produced from intron lariats, but the majority are entirely comprised of exons and arise from back-splicing, which links an exon to a preceding exon, or to itself in the case of single exon circRNAs (1). Two principal mechanisms cause the formation of such back-spliced circRNAs. The majority are believed to result from inverted repeats within introns, (typically Alu repeats), which base pair with each other to form a large hairpin that brings the back-spliced splice sites into proximity, with the spliceosome then catalysing the formation of the backsplice. These circRNAs are typically present at very low levels (2). Some circRNAs could be formed due to the binding of a dimeric RNA binding protein to introns flanking the backsplice junction, as observed with the alternative splicing regulators MBNL1 (3) and QKI (4). Since QKI and MBNL1 form homodimers (5,6), they can presumably bind to two distinct introns and thereby bring the flanking splice sites into proximity for backsplicing to occur.

While the extreme sensitivity of detection of circRNAs by short read sequencing has led to the identification of hundreds of thousands of different circRNAs, most of these are almost certainly accidents of mis-splicing, present at such low level that they are unlikely to be of functional consequence (2). However, a small proportion of the known circRNAs are present at much higher levels that are likely to be of functional significance. A few such circRNAs have indeed been shown to affect cell proliferation or phenotype (7), while some that do not yet have functions identified are directly regulated during cellular differentiation, consistent with a role in contributing to the cell phenotype (4).

We have chosen to investigate the mechanism of formation and the function of the DOCK1(2–27) circRNA because it is highly regulated during EMT. This unusually large circRNA of 2738 nt comprising exons 2–27 from the DOCK1 gene (with circBase designation hsa_circ_0020397) is expressed in epithelial cells but is strongly downregulated during epithelial to mesenchymal transition (EMT), while the DOCK1 mRNA is increased in level during EMT. We found that the formation of this circRNA is dependent on the splicing regulator ESRP1, which promotes circDOCK1(2–27) formation by inhibiting splicing of exon 1 to exon 2, thereby holding the intron 1 acceptor site unspliced and available while Pol II completes its 157 kb journey from exon 2 to exon 27. We found by HITS-CLIP analysis that ESRP1 binds to a GU-rich motif in a tandem repeat region in intron 1. We solved the crystal structure of ESRP1 qRRM2 bound to the repeat motif, revealing that the DOCK1 qRRM2 domain binds a GGU motif in a double clamp arrangement, with aromatic residues on either side of each G forming an aromatic pocket, while bound water molecules form hydrogen bonds to the uracil sidechain. The binding of ESRP1 to the intron 1 region is necessary and sufficient for the high-efficiency formation of the circDOCK1(2–27), so that splicing of the DOCK1 pre-mRNA is diverted to circRNA formation, limiting the production of DOCK1 mRNA and protein, while the circDOCK1(2–27) itself also directly reduces the migratory capacity of cells.

## Materials and methods

### Cell culture and transduction

Human cancer cell line culture conditions were described previously (8). Briefly, cell lines were cultured with 5% CO_2_ at 37°C. HEK293T, MCF-7, T47D, MDA-MB-361, ZR-75–1, MDA-MB-415, MDA-MB-134-VI, Hs578T and MDA-MB-231 were cultured in DMEM (Thermo Fisher) + 10% FCS. MDA-MB-436, MDA-MB-157, CAL51 and CAL120 cells were cultured in RPMI media with 10% FCS, 20 mM HEPES and 288 μl Insulin/100 ml. SUM159PT cells were cultured in Ham's F12 media with 5% FCS, 5 μg/ml insulin and 1 μg/ml hydrocortisone, HMLE cells in HuMEC basal serum free media (Thermo Fisher) and mesHMLE cells in Weinberg media (DMEM + F12 media with 5% FCS, 4 mg/ml insulin, 20 μg/ml EGF and 1 mg/ml hydrocortisone).

siRNAs were transfected with Lipofectamine RNAiMAX (Life Technologies) at 10 nM concentration following the manufacturer's protocol. DNA plasmid transfections were performed with Lipofectamine 2000 (Life Technologies) following the manufacturer's protocol.

### CRISPR knockout generation

A pair of lentiCRISPR v2 plasmids (a gift from Feng Zhang (Addgene plasmid # 52961) with designed sgRNAs flanking the target region were co-transfected with pcDNA3-GFP simultaneously into T47D and 293T cells. Three days after transfection, individual GFP positive cells were sorted into 96-well plates. PCR primers flanking the target region were designed and used to screen for individual clones with successful genomic deletion. To validate the genomic deletions by Sanger sequencing, 10 ng of purified PCR product and 10 pmol of sequencing primer were mixed and Sanger sequencing was performed by the Australian Genome Research Facility (AGRF).

### Recombinant DNA constructs

For CRISPR experiments, gRNAs were cloned into lentiCRISPR v2 (Plasmid #52961, Addgene) using the BsmBI site. For pINDUCER-20 (Plasmid #44012) doxycycline inducible gene expression, cDNA was firstly cloned into pENTR2B gateway entry vector, the pENTR2B derivative was then recombined with pINDUCER-20 using LR Clonase to produce doxycycline inducible expression. Viral production and transduction were performed as previously described (8). Generally, Lentivirus was produced in a T25 flask of HEK 293T cells beginning at 50–60% confluency. Plasmids encoding the gag-pol genes, the rev gene and the VSV-g envelope gene were co-transfected with pINDUCER-20 for viral production. After viral transduction, G418 was used for cell selection.

### Cell sorting

The optimal number of cells were resuspended into cell sorting buffer (5 mM EDTA, 25 mM HEPES, 2% fetal calf serum in 1× PBS). After filtering the cells through a 30 μm filter into a FACS tube, the cells were then sorted and collected by MoFlo Astrios Cell Sorter according to the manufacturer's protocol.

### Western Blotting

Cells were rinsed with cold 1× PBS then lysed on ice in 1× RIPA buffer (Abcam) supplemented with protease inhibitor cocktail (Roche) and phosphatase inhibitor (PhosSTOP, Roche). After centrifugation at 13 000 rpm for 20 min at 4°C, protein concentration was quantified by Bicinchoninic Acid assay (Pierce). Samples were diluted in 1× Bolt LDS sample buffer (ThermoFisher) supplemented with 2% beta-mercaptoethanol (Merck) final concentration. After denaturation at 70°C for 10 min, 10–20 μg of proteins were separated on Bolt bis-tris gel in 1× MOPS SDS running buffer (ThermoFisher) and transferred onto 0.45 μm nitrocellulose membrane (Advantec). Membranes were blocked in 5% skim milk in TBST buffer (20 mM Tris-HCl pH7.6, 150 mM NaCl, 0.2% Tween-20) for 1 h at room temperature then incubated with primary antibodies diluted in 3% BSA in TBST buffer. After 5 min wash with TBST buffer thrice, membranes were incubated with either HRP-conjugated (Pierce) or IRdye secondary antibodies (Licor) diluted in TBST buffer at 1:10 000. After 5 min wash with TBST buffer thrice, membranes were imaged using Chemidoc MP (Bio-Rad). Primary antibodies used in this study were: DOCK1 antibody (1:1000) from Bethyl Lab (A301-288), ESRP1 antibody (1:1000) from ThermoFisher (PA5-25833) and alpha-Tubulin (1:10000) from Abcam (ab7291). Secondary antibodies used in this study were: Goat anti-mouse IR680RD (LCR-926–68070), Goat anti-rabbit IR680RD (LCR-926-68071), Goat anti-rabbit IR800CW (LCR-926–32211), Goat anti-mouse-HRP (31430) and Goat anti-rabbit-HRP (31460).

### Migration assay

Transwell migration assays were performed as previously described (8). Generally, 2 × 10^5^ MDA-MB-231 or mesHMLE cells were plated into Transwells (Corning, 6.5 mm, 8.0 μm pore size) in serum-free medium, then 10% FCS was added into the lower chamber to induce chemotactic migration for 4 hours.

### RNA Isolation and PCR

RNA extraction, RT-PCR and qPCR were performed as previously described (8). TRIzol (ThermoFisher) was used for RNA extraction following the manufacturer's instructions. QuantiTect RT kit (Qiagen) was used for mRNA and circular RNA reverse transcription. The synthesised cDNA was then diluted 1:20 for quantitative PCR (qPCR). qPCR was performed in triplicate on a Rotor-Gene-Q series PCR machine (Qiagen) using the QuantiTect SYBR Green PCR kit (Qiagen). The sequences of primers are shown in Supplementary Table S4. Rotor-Gene software was used for data analysis and GAPDH expression was used for gene expression normalization.

Standard Taq polymerase (NEB) or Phusion DNA Polymerase (ThermoFisher) were used for standard PCR for gene clones.

### HITS-CLIP assay

HITS-CLIP was performed on endogenous ESRP1 from 3 biological replicates of wt HMLE cells using an ESRP1 specific antibody. We also performed HITS-CLIP using anti-FLAG antibody on 2 biological replicates each of N- and C-terminally 3XFLAG-tagged ESRP1 expressed in HMLE-i-ESRP1_FLAG cells after induction with 1 μg/ml doxycycline for 72 h. Cells grown to 80% confluency in 150mm dishes were rinsed with ice-cold PBS and UV irradiated twice with 300 mJ/cm2, 254 nm, in ice-cold PBS using a Spectrolinker XL1500 (Spectro-UV). Cells were lysed in the dish with 750 μl 1× PXL [1× PBS, 0.1% SDS, 0.5% deoxycholate, 0.5% Igepal] + EDTA-free Complete protease inhibitor cocktail (PIC; Roche), collected by scraping and stored at -80°C until use. Thawed lysates were triturated using a 21G needle and DNA digested with 40 μl Turbo DNAse (Ambion AM2238) at 37°C, 350 rpm for 10 min. RNA was partially digested with RNase 1 (Ambion AM2295) by adding 10 μl of 1:25 diluted RNase 1 per 1ml of lysate at 37°C for 5 min. Lysates were centrifuged at 21 000 g for 30 min at 4°C and supernatant transferred to a fresh tube.

ESRP1-RNA complexes were immunoprecipitated using rabbit polyclonal anti-ESRP1 antibody (Proteintech Cat# 21045-1-AP); 8 μg antibody pre-bound to to 75 μl protein A Dynabeads (ThermoFisher, 10002D), or mouse monoclonal anti-FLAG M2 antibody (Sigma Cat# F1804); 18 μg antibody pre-bound to to 75 μl protein G Dynabeads (ThermoFisher, 10004D). Negative controls were performed using wt HMLE lysates immunoprecipitated with rabbit IgG (ThermoFisher Cat# 02–6102), or anti-FLAG antibodies coupled to protein A or G beads respectively. Washed beads were resuspended with 1.0 ml of prepared lysate at ∼1.4mg/ml and rotated 75 min at 4°C. Bound ESRP1-RNA complexes were washed twice each consecutively with ice cold 1× PXL, 5× PXL [5× PBS, 0.1% SDS, 0.5% sodium deoxycholate, 0.5% Igepal], and 1× PNK [50 mM Tris–Cl pH 7.5, 10 mM MgCl_2_ and 0.5% Igepal]. Beads were first treated with T4 PNK (NEB, M0201L; 10 U in 80 μl reaction volume) in the absence of ATP (37°C, 850 rpm for 20 min in a thermomixer) to dephosphorylate 3′ RNA ends followed by washes with 1× PNK, 1× PNK + EGTA [50 mM Tris–Cl pH 7.5, 20 mM EGTA, and 0.5% Igepal], and two washes with 1× PNK at 4°C.

The 3′ preadenylated linker (NEBNext 3′SR adaptor for Illumina; /5rApp/AGA TCG GAA GAG CAC ACG TCT /3AmMO/) was ligated to the RNA fragments on bead using T4 RNA ligase 2 truncated KQ (NEB M0373) at 16°C, overnight with shaking. Beads were washed consecutively with ice cold 1× PNK, 5× PXL and twice with 1× PNK. Bound RNAs were then labelled with P32 γ-ATP using T4 PNK, 45 min at 37°C, followed by addition of 2.5 μM ATP, 5 mins at 37°C. Beads were washed twice each with ice-cold 1× PNK + EGTA and 1× PNK. The 5′ RNA linker (5′-blocked and containing a 10 nt UMI (/5AmMC6/GUUCAGAGUUCUACAGUCCGACGAUCNNNNNNNNNN3’) was ligated to the RNA fragments on bead using T4 RNA ligase (NEB M0437) for 1 hr at 25°C, with intermittent shaking. Beads were washed 3 times with ice-cold 1× PNK + EGTA.

Protein A–anti-ESRP1 and Protein A–IgG beads were eluted with 40 μl 1× Bolt LDS sample buffer (ThermoFisher) + 1% β-mercaptoethanol at 70°C for 10 min. Protein G–anti-FLAG beads were eluted with 30 μl 3× FLAG peptide (Sigma F4799; 250ug/ml in PBS, 0.02% Tween-20), 20°C for 30 min with shaking. 15 μl 4× Bolt LDS sample buffer + 4% β-mercaptoethanol was then added to the FLAG eluates and the samples heated at 70°C for 10 min. All samples were then separated through Bolt 8% Bis-tris Plus gels (ThermoFisher) using BOLT MOPS SDS running buffer at 175 V for 1 h. Protein-RNA complexes were then transferred to nitrocellulose (Schleicher&Schuell, BA-85) by wet transfer using 1× Bolt transfer buffer with 10% methanol. Filters were placed on a phosphor screen and exposed using a Typhoon imager (GE).

The 100–130 kDa region of each lane was excised, corresponding to ESRP1:RNA complexes with RNAs ∼20–100nt + linkers, and the RNA liberated by proteinase K digestion (2 mg/mL proteinase K, 100 mM Tris–HCl pH 7.5, 50 mM NaCl, 10 mM EDTA, 0.2% SDS) at 50°C for 60 min, 1200 rpm, followed by extraction with acid phenol (ThermoFisher, AM9712) and precipitation with 1:1 isopropanol:ethanol. RNA was pelleted by centrifugation then separated on an 8% denaturing polyacrylamide gel (1:19 acrylamide, 1– TBE, 7 M urea). The wet gel was wrapped in plastic wrap and exposed to a phosphor screen and imaged using a Typhoon. Gel slices were cut (size 75–150nt) and eluted by the ‘crush and soak’ method, followed by ethanol precipitation.

Reverse transcription was performed using a nested RT primer (IDT, AGACGTGTGCTCTTCCGA) with SuperScript IV and MnCl2 buffer [50 mM Tris pH 8.0, 75 mM KCl, 3 mM MnCl2] to enhance read-though at crosslink sites (9). Products were amplified for 12–16 cycles using NEBNext Ultra II Q5 mastermix (NEB cat#M0544) with a common forward primer (NEBNext SR primer for Illumina) and barcoded reverse primers for each sample (NEBNext Index primers for Illumina). PCR products were purified using 1.8 volumes of Axygen AxyPrep magnetic beads (MAG-PCR-CL), separated on an 8% acrylamide (19:1), 7 M urea TBE semi- denaturing gel, stained with SYBR Gold nucleic acid gel stain (ThermoFisher) and imaged on a ChemiDoc (BioRad). Products corresponding to an insert size of > 20 nt were excised from the gel and extracted by the ‘crush and soak’ method. Library quantity was determined by qPCR using NEBNext Library Quant kit for Illumina, pooled and sequenced on an Illumina NextSeq 500 (1 × 75 bp).

### HITS-CLIP bioinformatic analyses

The eleven HITS-CLIP libraries average raw sequencing depths of 79 million, 34 million, 36 million, 57 million and 4 million reads for the endogenous ESRP1, N-terminal FLAG-tagged inducible ESRP1, C-terminal FLAG-tagged inducible ESRP1, control IgG and control FLAG replicates, respectively. FASTQ files were analysed at various stages for quality and content using FastQC v0.11.9 (http://www.bioinformatics.babraham.ac.uk/projects/fastqc/) and raw reads were adapter trimmed and filtered using cutadapt v2.8 (10) using an adapter sequence of AGATCGGAAGAGCACACGTCTGAACTCCAGTCA, error rate of 0.2, and overlap of 5 and minimum length of 28. Reads derived from PCR duplication were collapsed using Unique Molecular Identifiers (UMIs) using UMI-tools (v0.5.3) (11) by first using the ‘extract’ method with default parameters to cut the 10 bp UMIs from the 3′ end of the reads. Reads were then mapped against the human reference genome (hg19) using the STAR (v2.7.2c) spliced alignment algorithm (12) with parameters –twopassMode basic and –quantMode GeneCounts and otherwise default parameters, at an alignment rate of ∼82–97%. Subsequently, unique molecular identifiers (UMIs) were used to collapse PCR duplicate reads using the UMI-tools ‘dedup’ method with default parameters. To identify enriched regions of the genome, replicate samples were pooled using the Picard Tools function MergeSamFiles (http://broadinstitute.github.io/picard/) and quality filtered using samtools (-q 10) (13).

For the endogenous ESRP1, N-terminal FLAG-tagged inducible ESRP1 and C-terminal FLAG-tagged inducible ESRP1, peak calling was then performed separately for each strand using MACS2 peak caller (version 2.1.1) (14) using the combination of the IgG and FLAG control samples as the control. The following settings were used (-f BAM -g hs –keep-dup all –nomodel –extsize 50 -B –call-summits –slocal 0 –llocal 0 –fe-cutoff 3 -q 0.05) and the resulting peak files from each strand were merged. HITS-CLIP peaks and alignments were visualized and interrogated using the Integrative Genomics Viewer v2.8.0 (15). Homer (16) was used to perform de novo motif enrichment analysis (findMotifsGenome.pl parameters: -size given -norevopp -len 5, 6, 7, 8, 9, 10). This identified several motifs similar to the published ESRP1 motif which were highly enriched; shown are motifs which were significantly enriched (*P*-value: <<1e-100) in the C-terminal FLAG samples.

### Relative intron abundance measurement

Relative RNA sequence read numbers in each DOCK1 intron were calculated using data from (8) and are deposited to the European Nucleotide Archive database (http://www.ebi.ac.uk/ena/data/view/PRJEB25042) with the study accession number PRJEB25042. We first determined the number of reads mapping to each intron in a strand-specific manner, counting only reads with the read start inside the intron. The two smallest introns, intron 34 and intron 41, both less than 200 bp, were discarded as they contained too few reads to be accurately quantified. The counts for each intron were then normalised by the length of that intron, adding a pseudocount of 1 and transforming the data by log2. Note that for DOCK1 intron 1, the iGenomes hg19 genome assembly version used contains a large region of low complexity which is masked (represented as ‘N’s). This region was excluded from the analysis. To remove the influence of differences in DOCK1 gene expression, we subtracted the expression of the gene in that sample (defined as the median intron coverage per kb) from the values calculated above. To determine whether reads were statistically significantly overrepresented in the HMLE intron 1 relative to (a) the MesHMLE intron 1, or (b) other introns in the same cell line, we used a students’ t-test with multiple-testing correction using the Bonferroni method. We used two biological replicates for HMLE samples and three for MesHMLE samples.

### Protein purification

The cDNA for human ESRP1 (amino acids 1–681, UniProt ID: Q6NXG1) was purchased from Shanghai Generay Biotech Co., Ltd, China. The target fragment of qRRM2 (312–430) was amplified by PCR reaction and cloned into a modified pET28a-SUMO vector, the recombinant vector was then transferred into Escherichia coli BL21 (DE3) competent cell for protein expression. The plasmids of ESRP1 mutants were obtained by overlap PCR using the wild-type ESRP1 plasmid as the template. Sequences of wildtype and all mutant plasmids were confirmed by DNA sequencing. The frozen recombinant strains were cultivated at 37°C in LB medium supplemented with 50 μg/mL kanamycin. The protein expressions were induced at OD_600_ of 0.6–0.8 by adding of isopropyl β-d-1-thiogalactopyranoside (IPTG) at a final concentration of 0.2 mM. The cultures were incubated at 18°C for an additional 16 h to allow the accumulation of expressed proteins. Cells were harvested by centrifugation, resuspended in buffer 1 (20 mM Tris–HCl pH 8.0, 500 mM NaCl, 25 mM imidazole pH 8.0), and lysed under high pressure. Cell extracts were centrifuged at 18000 rpm for 1h at 4°C. Supernatants were applied to a HisTrapTM HP column pre-equilibrated with buffer 1, and the target proteins were eluted from the column using buffer 2 (20 mM Tris pH 8.0, 500 mM NaCl, 500 mM imidazole pH 8.0) with a gradient. The recombinant protein was dialyzed against buffer S (20 mM Tris pH 8.0, 500 mM NaCl) for 3 hours, Ulp1 protease was added to remove the His-Sumo tag. The mixture was applied to HisTrapTM HP column again and the fractions containing the target protein were pooled, concentrated and loaded onto a HiLoadTM 16/600 Superdex 75 column pre-equilibrated using gel filtration buffer (10 mM Tris pH 8.0, 100 mM NaCl). Purities of the proteins were analyzed using SDS-PAGE gel and the samples were stored at –80°C until use. Protein concentration was determined using UV observation at A280.

### Crystallization and data collection

Crystals were grown using the sitting drop vapor diffusion method at 20°C with the drop composed of 0.5 μl of protein-RNA sample and 0.5 μl of crystallization solution. The qRRM2-RNA complex crystals suitable for X-ray diffraction were grown in reservoir solution consisting of 0.2 M Sodium malonate pH 7.0, 20% w/v Polyethylene glycol 3350. Crystals were cryoprotected using their mother liquor supplemented with 25% glycerol and snap-frozen in liquid nitrogen. X-ray diffraction data were collected on beamline BL19U1, BL17U1 and BL18U1 at the Shanghai Synchrotron Radiation Facility (SSRF). Data processing was carried out using the HKL3000 programs or XDS (17,18). The data collection and processing statistics are summarized in Supplementary Table S3.

### Structure determination and refinement

The qRRM2-RNA complex structure was determined by molecular replacement (MR) method using the Phaser program embedded in the CCP4i suite (19), the structure of the protein (PDB code: 2RVJ) was used as the search model (20). The resulting model was refined against the diffraction data using the REFMAC5 program (21) in CCP4i and the model building was performed using the program COOT (22). The 2Fo-Fc and Fo-Fc electron density maps were regularly calculated and used as guides for model building. The final refinement of structure was done using the phenix.refine program (23) of PHENIX. Ramachandran analysis showed that all the protein residues are located in the most favored or allowed region. The detailed structural refinement statistics are summarized in Supplementary Table S3. All structure figures were prepared with PyMOL (DeLano Scientific).

### Isothermal titration calorimetry

ITC titrations were performed at 25°C using a MicroCal PEAQ instrument with titration buffer composed of 100 mM NaCl and 10 mM Tris-HCl pH 8.0. The concentrations of proteins were determined spectrophotometrically. The RNA substrates were diluted in the reaction buffer. The ITC experiments involved 20 or 25 injections of protein into RNA. The sample cell was loaded with 300 uL of RNA at 20 μM and the syringe with 80 ml of protein at 300 μM. Curve fitting to a single binding site model was performed using the ITC data analysis module of Origin 7.0 (MicroCal) provided by the manufacturer. Δ*G°* of protein–RNA binding was computed as *RT*ln(1/*K*_d_), where *R*, *T* and *K*_d_ are the gas constant, temperature and dissociation constant, respectively.

### BioID samples

The ESPR1 Open Reading Frame (corresponding to NP_060167.2, from the ORFeome 8.1 collection in pDONR223) was cloned using GatewayTM enzymes in the pDEST-pcDNA5-C-term-FLAG-BirA* vector, adding a FLAG and BirA* fusion to the C-terminus of ESPR1. This construct was transfected (along negative controls consisting of the parental BirA*-FLAG tag) into Flp-In T-REx 293 cells (Invitrogen), and pools of stable transfectants were selected (internal reference C2982) and processed for BioID (alongside an additional negative control consisting of non-transfected cells), essentially as in (24) with minor modifications. Harvested cells were lysed in modified RIPA buffer [50 mM Tris (pH 7.5), 150 mM NaCl, 1.5 mM MgCl_2_, 1 mM EGTA, 0.1% SDS, 1% IGEPAL CA-630] with freshly added sodium deoxycholate (0.4%) and protease inhibitors (Sigma-Aldrich P8340) at 400 μl/0.1 g cells, sonicated, and treated benzonase (375 U per sample) for 15 min at 4°C. SDS was adjusted to a final concentration of 0.4% (incubation 15 min, 4°C). Samples were centrifuged (16 000 × g for 20 min) and the cleared lysates were incubated with pre-washed streptavidin Sepharose beads (GE 17-5113-01; 30 μl bed volume) for 3 h, 4°C. Beads were washed once with wash buffer [2% SDS, 50 mM Tris (pH 7.5)], 2× with modified RIPA buffer with 0.4% SDS, and 3× with ABC buffer [50 mM ammonium bicarbonate (pH 8.5)]. Beads were pelleted, supernatant removed, and on-bead trypsin digest of peptides was performed in two-steps (1 μg trypsin overnight; 0.5 μg trypsin for 2 h; both at 37°C). Supernatants were transferred to new tubes (beads were rinsed twice in water and supernatants combined). Freshly made 50% formic acid was added to samples to a final concentration of 2% prior to drying by vacuum centrifugation and storage at –80°C.

### Mass spectrometry acquisition and analysis

Each sample (6 μL in 2% formic acid; corresponding to 1/6th of a 15 cm tissue culture dish) was directly loaded at 800 nL/min onto an equilibrated HPLC column (pulled and packed in-house). The peptides were eluted from the column over a 90 min gradient generated by a Eksigent ekspert™ nanoLC 425 (Eksigent, Dublin CA) nano-pump and analysed on a TripleTOF 6600 instrument (AB SCIEX, Concord, Ontario, Canada). The Data Dependent Acquisition method consisted of one 250 milliseconds (ms) MS1 TOF survey scan from 400 to 1800 Da followed by ten 100 ms MS2 candidate ion scans from 100 to 1800 Da in high sensitivity mode. Only ions with a charge of 2+ to 5+ that exceeded a threshold of 300 cps were selected for MS2, and former precursors were excluded for 7 s after one occurrence. Data were stored, searched and analyzed using ProHits laboratory information management system (25). Within ProHits, WIFF files were converted to an MGF format using the WIFF2MGF converter and to an mzML format using ProteoWizard (V3.0.10702) and the AB SCIEX MS Data Converter (V1.3 beta). The data was then searched using Mascot (V2.3.02) (26) and Comet (V2016.01 rev.2) (27). The spectra were searched with the human and adenovirus sequences in the RefSeq database (version 57, 30 January 2013) acquired from NCBI, supplemented with ‘common contaminants’ from the Max Planck Institute (http://maxquant.org/contaminants.zip) and the Global Proteome Machine (GPM; ftp://ftp.thegpm.org/fasta/cRAP/crap.fasta), forward and reverse sequences (labeled ‘gi|9999″ or ‘DECOY’), sequence tags (BirA, GST, mCherry and GFP) and streptavidin, for a total of 72481 entries. Database parameters were set to search for tryptic cleavages, allowing up to 2 missed cleavages sites per peptide with a mass tolerance of 35 ppm for precursors with charges of 2+ to 4+ and a tolerance of 0.15 amu for fragment ions. Variable modifications were selected for deamidated asparagine and glutamine and oxidized methionine. Results from each search engine were analyzed through TPP (the Trans-Proteomic Pipeline, v.4.7 POLAR VORTEX rev 1) via the iProphet pipeline (28), and only proteins with a 95% FDR iProphet filter were considered further. SAINTexpress version 3.6.1 was used as a statistical tool to calculate the probability of potential protein-protein associations compared to background contaminants using default parameters, and control compression set to 2 (effectively compressing the four negative controls to two virtual controls) (29). SAINT scores with a Bayesian false discovery rate (BFDR) ≤ 1% were considered high-confidence protein interactions. All non-human protein interactors (did not start with ‘NP’ in Prey column) were removed from the SAINT analysis, except for BirA_R118G_H0QFJ5. Visualization and additional analysis of the data was through ProHits-viz.org (30) and the humancellmap.org (31) resources, using default options. Enrichment was performed using g:profiler (32) with default options.

### Statistical analyses

Unless otherwise stated, data are shown as the mean of three replicate experiments ± SEM, with statistically significant *t*-tests shown as * *P* < 0.05, ** *P* < 0.01 or *** *P* < 0.001.

## Results

### Formation of the DOCK1(**2**-**27**) circRNA is regulated by TGF-β and downregulated in mesenchymal tumours

**Figure 1.**
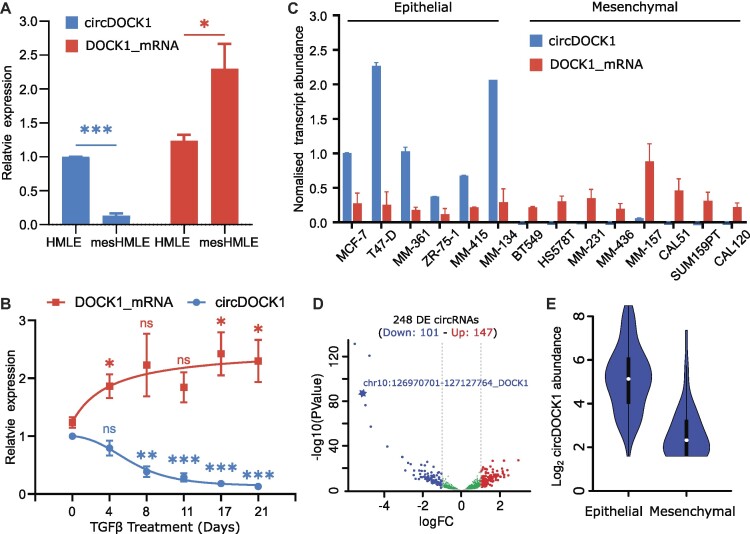
circDOCK1(2–27) is epithelial-specific and regulated during EMT. (**A**) circDOCK1(2–27) and DOCK1 mRNA quantitation from qPCR of RNA from HMLE cells before and after prolonged treatment with TGF-β. Mean ± SEM. *n* = 3 biological replicates, each performed in technical triplicate. * *P* < 0.05, *** *P* < 0.001, Student's *t*-test, two-tailed. The qPCR Ct values are given in Supplementary Figure S1A. (**B**) Time course of circDOCK1(2–27) and DOCK1 mRNA levels in TGF-β-treated HMLE cells measured by qPCR. (**C**) circDOCK1(2–27) and DOCK1 mRNA levels measured by qPCR in epithelial and mesenchymal breast cancer cell lines. The data are all expressed relative to the level of the circRNA in MCF7 cells. **(D)** Differential expression analysis of 4095 high confidence circRNAs in epithelial versus mesenchymal tumors using data from MiOncoCirc (54). Tumor samples were in-silico classified into epithelial or mesenchymal groups based on the parental gene expression of eight EMT marker genes (epithelial markers; CDH1, ESRP1, ESRP2 and CLDN7—mesenchymal markers; CDH2, VIM, ZEB1 and ZEB2) (Supplementary Figure S1E). (**E**) CircDOCK1 expression in the 191 epithelial and 159 mesenchymal primary tumor samples classified as in (D).

Motivated by the observation in our previous study of regulated changes in abundance of circRNAs during EMT that a circRNA arising from the DOCK1 gene appears to be regulated in the opposite direction from QKI-regulated circRNAs (4), we performed qPCR to measure circDOCK1(2–27) and DOCK1 mRNA in RNA extracted from human breast epithelial cells (HMLE cells) before and after prolonged treatment with TGF-β (producing mesenchymal ‘mesHMLE’ cells). This confirmed that the large circRNA from the DOCK1 gene, comprised of exons 2–27, which we call circDOCK1(2–27), was strongly downregulated following the TGF-β treatment (Figures 1A and S1A, B). Interestingly, the DOCK1 mRNA did not decrease, but instead was increased, suggesting that the TGF-β was repressing biogenesis of the circRNA rather than repressing transcription of the DOCK1 gene. To confirm this regulation of the circDOCK1(2–27) by TGF-β, we monitored its expression in HMLE cells during a time course of treatment with TGF-β and found the circRNA level progressively reduced over the 21 day period, while the DOCK1 mRNA level progressively increased (Figure 1B), similar to the mesenchymal gene ZEB1 (Supplementary Figure S1D). To check whether the epithelial-specific expression of the circDOCK1(2–27) circRNA is a general feature of breast cancers, we measured its expression in a panel of breast cancer cell lines, and also calculated its expression in epithelial versus mesenchymal tumours of diverse lineage, which confirmed its highly epithelial-specific expression (Figure 1C–E and Supplementary Figure S1C, E).

### Formation of the DOCK1(**2**-**27**) circRNA is dependent on ESRP1

**Figure 2.**
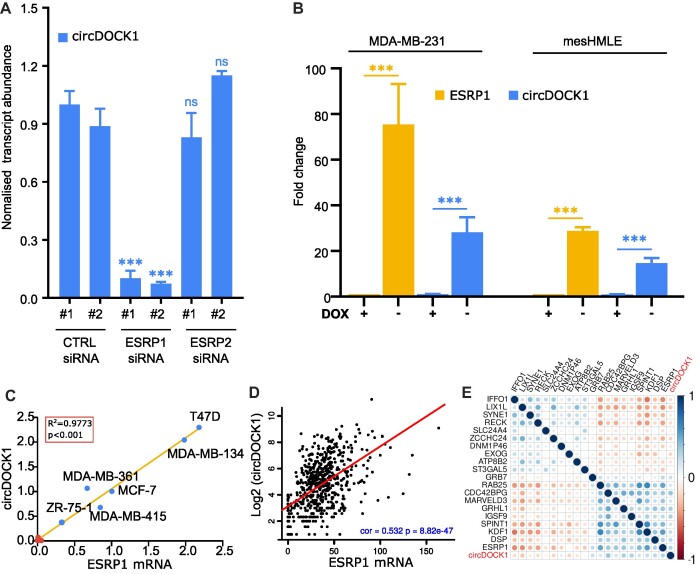
ESRP1 regulates the circDOCK1(2–27) level. (**A**) The expression of circDOCK1(2–27) was measured by qPCR in HMLE cells after siRNA knockdown of ESRP1 and ESRP2 with each of two independent siRNAs. (**B**) qPCR measurement of ESRP1 mRNA and circDOCK1(2–27) in MDA-MB-231 and mesHMLE cells transduced with dox-regulated ESRP1, before and after dox-mediated induction of ESRP1 for 3 days. (**C**) Relative levels of ESRP1 mRNA and circDOCK1(2–27) in the breast cancer cell lines shown in Figure 1C. (**D**) The correlation between ESRP1 mRNA and circDOCK1(2–27) in primary tumor samples, obtained using data from the miOncoCirc database (54). *R* = 0.532, *P*= 8.8e-47. (**E**) The mRNA transcripts most strongly correlated with circDOCK1(2–27) in data from miOncoCirc are shown. The colour intensity represents the correlation coefficient R and the circle size is proportional to the absolute value of R. The 10 most positively and 10 most negatively correlated transcripts with *P*< 1e-10 are shown. Data are from all tumours in the miOncoCirc database (54). The full data table is shown as Supplementary Table S1.

Given its epithelial-specific expression, we hypothesised that the formation of circDOCK1(2–27) may be regulated by an epithelial-specific splicing factor and so we asked whether its level is affected by depletion of either ESRP1 or ESRP2, which are well-known epithelial-specific splicing regulators (33). Depletion of ESRP1 by either of two independent siRNAs (Supplementary Figure S2A) drastically reduced the level of circDOCK1(2–27) in HMLE cells (Figure 2A), whereas depletion of ESRP2, which is present at 40% the level of ESRP1 (Supplementary Figure S2), had no effect on circDOCK1(2–27) (Figure 2A). To assess whether introducing ESRP1 into mesenchymal cells was sufficient to drive the formation of the circRNA, we constructed dox-inducible ESRP1 lentivirus and expressed ESRP1 in mesenchymal MDA-MB-231 and mesHMLE cells. ESRP1 induction caused a large increase in the level of circDOCK1(2–27) in each of these cell lines (Figure 2B). Consistent with these observations, the decline of ESRP1 in TGF-β-treated cells is matched by a decline in circDOCK1(2–27) level (Supplementary Figure S2C). Moreover, circDOCK1(2–27) levels are strongly correlated with the level of ESRP1 in breast cancer cell lines and in cancers in general (Figure 2C and D), and circDOCK1(2–27) is more strongly correlated with ESRP1 than with any other transcript in cancers in general (Figure 2E and Supplementary Table S1). Together these data indicate that ESRP1 potently regulates the formation of circDOCK1(2–27).

### DOCK1 circRNA formation competes with linear splicing, reducing DOCK1 mRNA and protein and affecting cell migration

**Figure 3.**
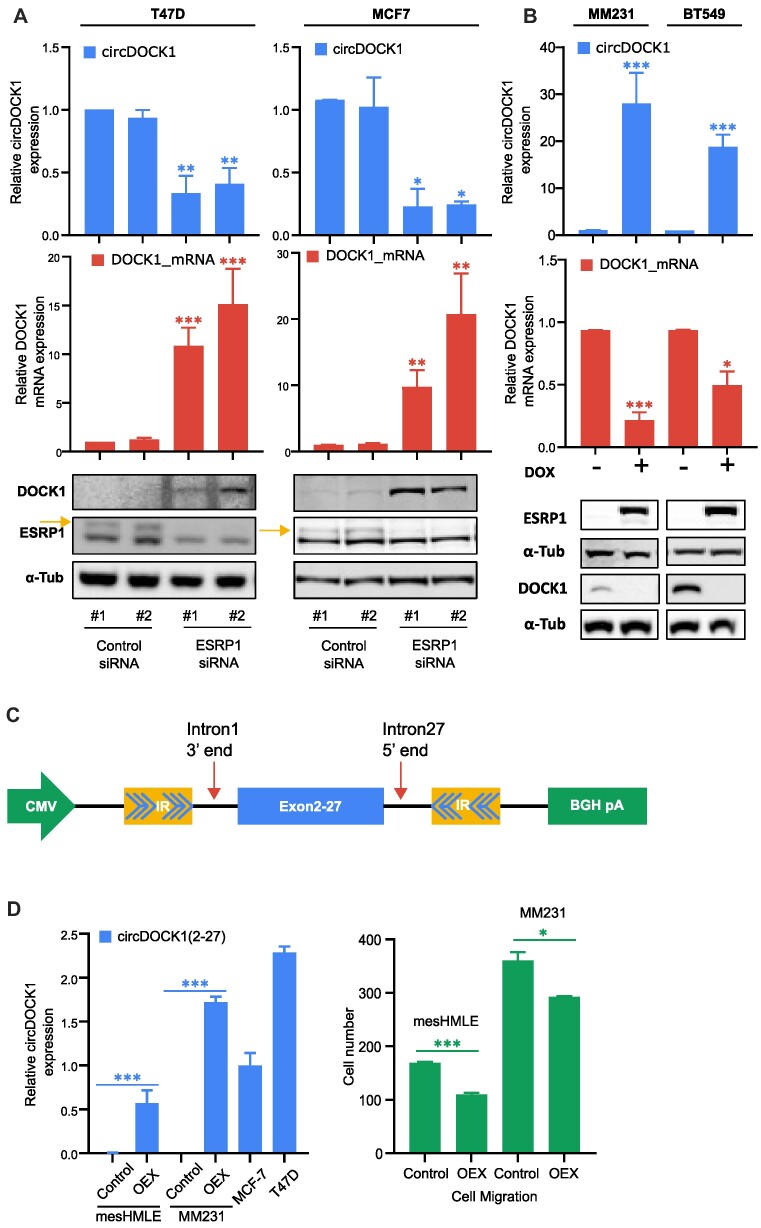
Effect of ESRP1 on DOCK1 expression and function. (**A**) qPCR of circDOCK1(2–27) and DOCK1 mRNA and immunoblot of DOCK1 and ESRP1 protein in epithelial cells transfected with control siRNAs or siRNAs to ESRP1. (**B**) qPCR of circDOCK1(2–27) and DOCK1 mRNA and immunoblot of DOCK1 and ESRP1 protein in mesenchymal cells with and without doxycycline induction of ESRP1. (**C**) Schematic of construct for expressing circDOCK1(2–27) in transfected cells, showing inverted repeat regions (IR) inserted into the DOCK1 intron regions. The downstream half of the inverted repeat was made by inverted insertion of a segment from intron 1. (**D**) Effect of circDOCK1(2–27) expression, measured by qPCR (left panel) on migration of mesenchymal cells measured by Transwell assay (right panel).

The apparently reciprocal relationship between DOCK1 mRNA and circRNA in epithelial versus mesenchymal cells (Figure 1) raises the possibility that channelling of pre-mRNA into the circRNA form in epithelial cells contributes to reducing DOCK1 mRNA and protein levels. To assess this, we first examined whether depletion or overexpression of ESRP1 affects DOCK1 levels. Indeed, knockdown of ESRP1 increased the level of DOCK1 mRNA and protein in epithelial cells (Figure 3A), while enforced expression of ESRP1 in mesenchymal cells decreased the level of DOCK1 mRNA and protein (Figure 3B), indicating that the formation of circDOCK1 competes substantially with the production of DOCK1 mRNA. Consequently, the DOCK1 protein is more abundant in mesenchymal cells than in epithelial cells (Supplementary Figure S3A).

Since DOCK1 is well known to be a promoter of cell migration (34), a reduction in DOCK1 protein level is likely to reduce the migratory capacity of cells. With this in mind, we were also interested in determining whether the DOCK1 circRNA itself can have a direct effect on cell migration, in addition to its indirect effect via modulation of DOCK1 protein, so we assessed the effect of artificial overexpression of the circDOCK1(2–27) on cell migration. To do this, we constructed a circDOCK1(2–27) expression vector by incorporating the cDNA for the circRNA flanked by splice sites and inverted repeats in the intron regions to promote circularisation (Figure 3C). We confirmed that the expression vector gives rise to predominantly circRNA by performing qPCR assessment of linear and circRNA forms of the transcript (Supplementary Figure S3B, C). Enforced expression of the circDOCK1(2–27) in mesenchymal cells at levels comparable to those in epithelial cells reduced the cell migration rate (Figure 3D and Supplementary Figure S3D). Thus the formation of circDOCK1(2–27) has a two-fold effect on the migratory capacity of cells, acting both directly to limit migration, and indirectly by reducing the level of DOCK1 protein production.

### HITS-CLIP analysis and crystal structure determination show ESRP1 binds to GGU sequences within the DOCK1 intron 1

**Figure 4.**
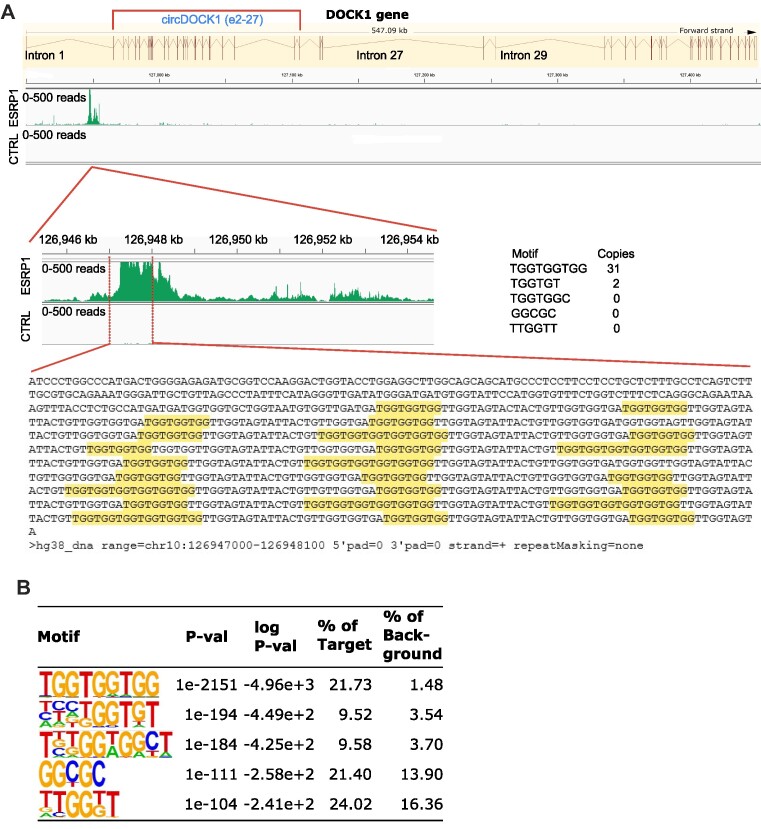
ESRP1 regulates formation of circDOCK1 via binding sites in intron 1. (**A**) Schematic showing the location of large peaks of ESRP1 interaction in DOCK1 pre-mRNA, with the major peak centred on a region that has 31 copies of the UGGUGGUGG motif. (**B**) ESRP1 binding motif identified by ESRP1 HITS-CLIP. De novo motif enrichment analysis was performed using HOMER(16) with parameters (findMotifsGenome.pl parameters: -size given -norevopp -len 5, 6, 7, 8, 9, 10).

To assess whether ESRP1 is directly involved in the biogenesis of the circDOCK1(2–27), we asked whether ESRP1 binds to DOCK1 pre-mRNA by performing HITS-CLIP analysis in HMLE cells. We observed a large peak of ESRP1 binding in intron 1, 23 kb upstream of the 3′ splice site (Figure 4A). Motif search analysis of ESRP1 binding peaks across the transcriptome produced a preferred motif of UGGUGGUGG, although several other G-rich motifs were also abundant (Figure 4B). The large binding peak within DOCK1 intron 1 covered a 1.2 kb region that contains 31 copies of this motif, as well as a nearby broad set of peaks spanning 4 kb with 21 additional copies of the UGGUGGUGG motif (Figures 4A and Supplementary Figure S4). The other motifs from the global motif search were absent or very low in number in these intron 1 regions.

**Figure 5.**
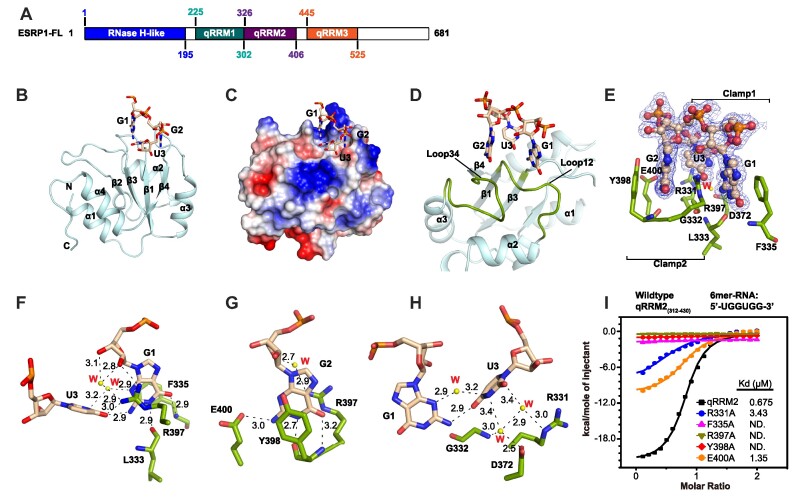
Crystal structure of the ESRP1 RRM2-RNA complex. (**A**) Domain organization of ESRP1. (**B**) The overall structure of ESRP1 qRRM2 with RNA. The qRRM2 is shown as ribbon and the RNA strand is shown as stick form. (**C**) Surface representation of the ESRP1 qRRM2-RNA complex with positive charged regions in blue and negative in red. (**D**) The RNA binding region in qRRM2 is coloured by green and indicated as Loop12 and Loop34. (**E**) Overall interactions between qRRM2 and RNA. The F_o_-F_c_ electron density contour level is 1.5 σ at 1.65 Å resolution. (**F–H**) Detailed interactions between qRRM2 and the 5′-GGU-3′ motif. (**I**) Isothermal titration calorimetry (ITC) of wildtype qRRM2 and its mutants with 6mer-RNA substrate (5′-UGGUGG-3′).

Because the global motif search of the ESRP1 HITS-CLIP peaks produced multiple candidate binding motifs, we sought to clarify the RNA sequence that ESRP1 binds by X-ray crystallography of the protein–RNA complex. The full-length ESRP1 is predicted to contain an RNase H-like domain and three quasi-RNA-recognition motifs (qRRMs) similar to those in hnRNP F (35) (Figure 5A and Supplementary Figure S5A). Crystals that diffracted to 1.65A with space group P3_1_2_1_ were obtained for the ESRP1 qRRM2 domain complexed to a 12-mer RNA with sequence UGGUGGUGGUGG. The qRRM2 comprises four β-stands flanked by two alpha-helices at each side of the β-sheet (Figure 5B). These helixes cover the beta-sheet region, making the qRRM domain different from canonical RRM domains, in which the RNA-binding region is located at the beta-sheet region, whereas the qRRM2 of ESRP1 binds to the RNA substrate through loop regions (Figure 5B). The structure shows contacts with just three nucleotides of the RNA, with the sequence GGU binding within a double clamp arrangement (Figure 5B–E). The three nucleotides are accommodated into a positively charged region (Figure 5C), with two loop regions important for nucleotide binding, one of which is between β1 and α2 (Loop12 in Figure 5D) and another one between α3 and β4 (Loop34). The three nucleotide bases face down to Loop12 and Loop34, and the phosphate groups of the RNA are distal from the protein backbone, enhancing the sequence-specific RNA recognition by qRRM2 (Figure 5D,E).

A close view of the interactions reveals that the two guanine residues are inserted into two clamps separated by Arg397 and form a **π–π** stacking like a sandwich (Figure 5E). For G1 recognition, besides the stacking interactions from Arg397 and Phe335, the base of G1 is also specifically recognized through a number of hydrogen bonds (Figure 5F). The O6 position is bound by the main-chain amino group of Phe335 and the N1 position is recognized by the main-chain of Leu333 (Figure 5F). The sugar group of G1 also contacts with the side chain of Arg397. Both the 2′-OH and the 3′-OH are hydrogen-bonded with the side chain of Arg397 directly or mediated by water. G2 is clamped by the side chain of Arg397 and Tyr398 (Figure 5G). The base of G2 is recognized by the main chain of Tyr398 and the side chain carboxyl group of Glu400 (Figure 5G). For U3, residues including Arg331, Gly332 and Asp372 donate hydrogen contacts mediated by water molecules, moreover, the U3 is stabilized through interacting with the G1 base (Figure 5H). The N2 position of G1 forms a hydrogen bond with the O4 of U3, which is also directly bound by the side chain of Arg397, and the N3 position of U3 interacts with the N3 position of G1 via a hydrogen bond mediated by a water molecule (Figure 5F,H). The interactions between the qRRM2 and RNA were validated by isothermal titration calorimetry (ITC) experiments. Mutating each of the three important residues Phe335, Arg397 and Tyr398 to alanine resulted in the loss of RNA binding ability, and the R331A and E400A mutants showed dramatically decreased RNA binding affinity, consistent with the observations in the complex structure (Figure 5I).

Given that all the residues making these bonds are conserved in the ESRP1 qRRM1 and qRRM3 domains (Supplementary Figure S5B), we anticipate that those domains also bind GGU triplets, making the optimal overall binding sequence for ESRP1 potentially GGU(N_x_)GGU(N_y_)GGU.

### Binding of ESRP1 detains splicing of DOCK1 intron 1

**Figure 6.**
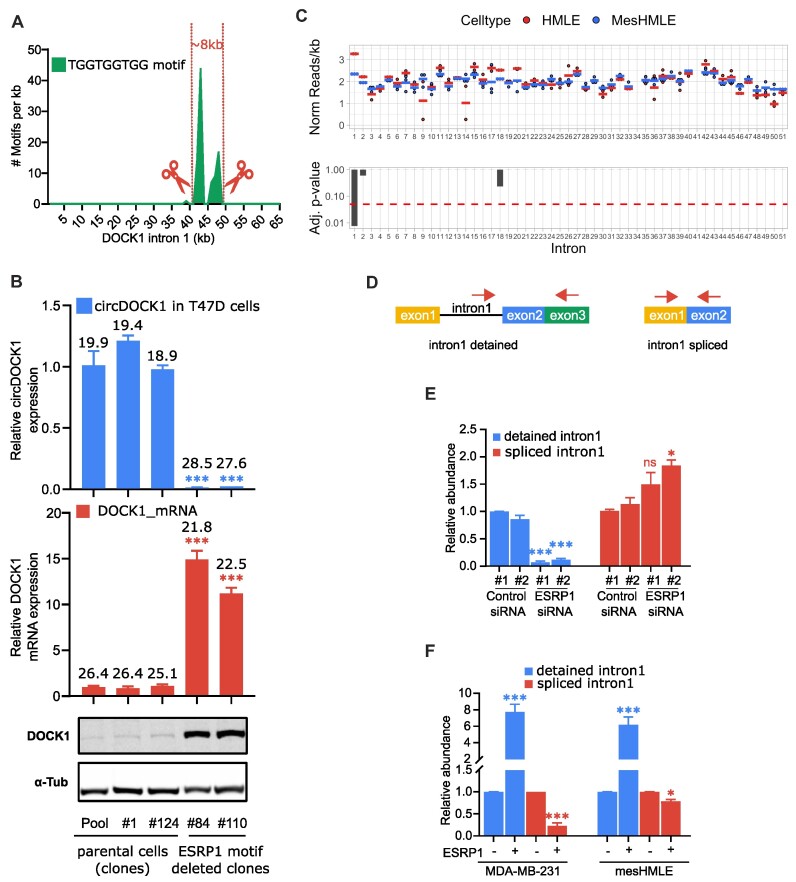
ESRP1 detains DOCK1 Intron 1 to facilitate circDOCK1 back splicing. (**A**) Plot of the occurrence of UGGUGGUGG motifs within intron 1, with the sites for CRISPR/Cas9-mediated deletion indicated. (**B**) The relative level of circDOCK1(2–27), DOCK1 mRNA and DOCK1 protein in parental T47D cells and in clones that have the 8kb region encompassing the ESRP1 binding peaks deleted. (**C**) Comparison of RNA sequence read numbers in each DOCK1 intron in HMLE and mesHMLE cells. The upper panel shows the average normalised read coverage for each intron from three RNA seq experiments, while the lower panel shows the significance level of the difference between coverage in HMLE versus mesHMLE cells. Only intron 1 has a large and significant difference between the cell lines. (**D**) Schematic diagram of qPCR primers detecting detained DOCK1 intron 1 RNA, with intron 2 spliced out but intron 1 remaining unspliced (left schematic) and DOCK1 RNA with intron 1 spliced out (right schematic). (**E**) Effect of ESRP1 depletion on intron 1 detention. The relative level of detained intron 1 (left panel) and spliced DOCK1 RNA (right panel) in shown for HMLE cells transfected with each of two control siRNAs or two ESRP1 siRNAs. (**F**) Effect of doxycycline inducible expression of ESRP1 in MDA-MB-231 and mesHMLE cells. MDA-MB-231 and mesHMLE cells stably transduced with pInducer lentivirus carrying dox-inducible ESRP1 were induced for 3 days and the change in level of detained intron 1 and spliced DOCK1 RNA measured by qPCR.

To determine whether the ESRP1 binding region in intron 1 participates in controlling the formation of the circDOCK1(2–27), we used CRISPR/Cas9 to delete 8 kb of the intron encompassing the two ESRP1 peaks (Figure 6A and Supplementary Figure S6A) and examined the effect on circDOCK1(2–27) levels. Deletion of this ESRP1 binding region almost completely eliminated expression of the circRNA (Figure 6B). ESRP1 mRNA levels were unaffected by this deletion, as expected (Supplementary Figure S6B). These results indicate that the ESRP1 binding region within intron 1 is essential for formation of the DOCK1(2–27) circRNA. Furthermore, the deletion of the ESRP1 binding region in intron 1 caused a large increase in DOCK1 mRNA and protein (Figure 6B), consistent with the backsplicing to form circDOCK1(2–27) in epithelial cells diverting product from mRNA to circRNA.

Having established that ESRP1 binding within intron 1 controls the production of circDOCK1(2–27), we sought to understand how ESRP1 binding exerts this effect. Mindful that the formation of the circRNA requires intron 1 to remain unspliced until transcription has proceeded to exon 27, we considered whether one function of ESRP1 may be to detain intron 1 in the unspliced form long enough to allow PolII to transcribe the 157 kb from exon 2 to exon 27, which is expected to take at least 40 minutes (36). First, we examined our RNA seq data to assess whether the relative level of intron 1, compared to other DOCK1 introns, is higher in epithelial cells (which express ESRP1), than in mesenchymal cells (which do not express ESRP1). We found that the relative level of intron 1 was increased substantially in HMLE cells compared to the relative level in mesHMLE cells, with *P*< 0.01 (Figure 6C), consistent with a reduced rate of intron 1 splicing in the epithelial cells (which we call ‘intron detention’). To further confirm this, we devised a qPCR assay for detained intron 1, amplifying from within intron 1 to exon 3, thereby requiring the 7 kb intron 2 to be spliced out for the PCR to be productive (Figure 6D). We compared the level of this splicing intermediate (intron 1 present, intron 2 removed) to the level of DOCK1 RNA with intron 1 removed, in the presence and absence of ESRP1. Depletion of ESRP1 in HMLE cells caused a large decrease in the level of detained intron 1 and increased the level of spliced RNA (Figure 6E). In the complementary experiment, introducing ESRP1 into mesenchymal cells resulted in an increased level of detained intron 1 and a decrease in spliced DOCK1 RNA with intron 1 removed (Figure 6F). These experiments demonstrate that ESRP1 detains the splicing of DOCK1 intron 1.

### Potential mechanisms of DOCK1 circRNA formation

**Figure 7.**
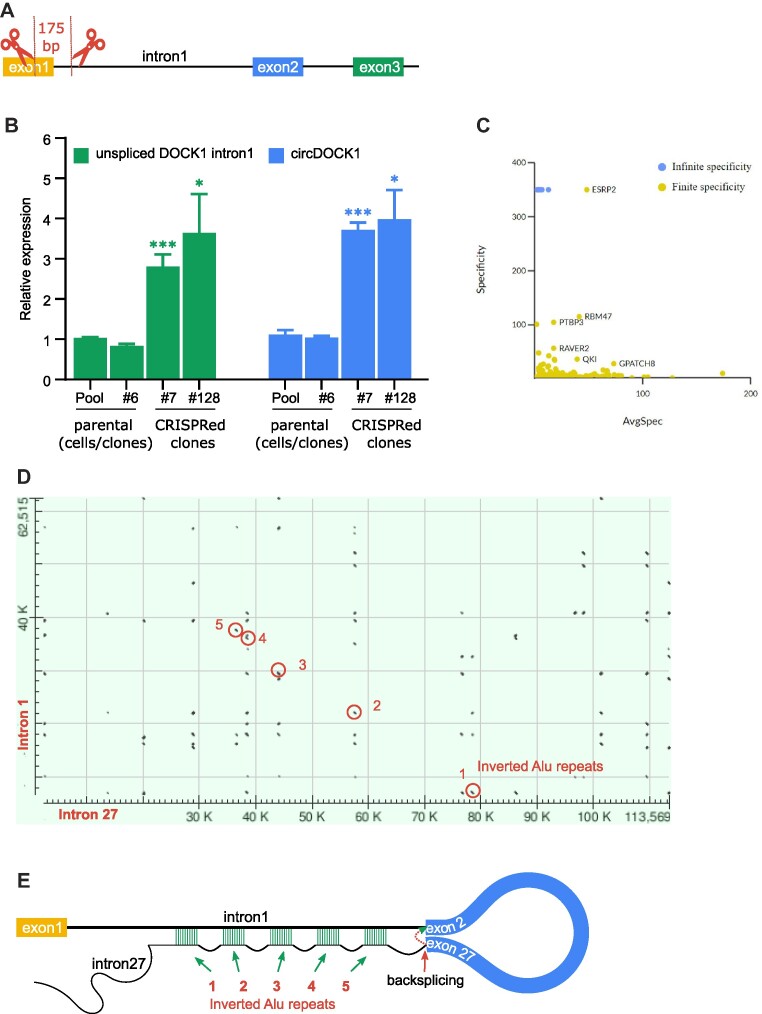
Intron 1 detention promotes circDOCK1(2–27) formation. (**A**) Schematic diagram of CRISPR/Cas9 cut sites removing the intron 1 5′ splice site. (**B**) The relative level of detained intron 1 (green) and circDOCK1(2–27) (blue) measured by qPCR in HEK293T cells and in two independent clones that had the intron 1 splice site deleted. (**C**) Scatterplot showing the most specifically enriched proximal interactions for ESRP1 when compared against all baits in the humancellmap.org. (**D**) Alignment of intron 1 sequence with intron 27, generated from NCBI blastn. Selected Alu inverted repeats that might give rise to base pairing interactions are circled. (**E**) Schematic of potential base pairing between Alu repeats in DOCK1 intron 1 and intron 27.

We next asked whether the role of ESRP1 is simply to detain intron 1, or whether it has additional roles in promoting circRNA formation. If intron 1 detention is alone sufficient to promote the formation of the circDOCK1(2–27), then artificially detaining intron 1 splicing should promote circRNA formation even in the absence of ESRP1. To prevent intron 1 splicing we used CRISPR/Cas9 to remove the intron 1 5′ splice site, but leaving the bulk of the intron and its 3′ splice site intact (Figure 7A). This was done in HEK293 cells, which express minimal ESRP1 (<0.1% of the level expressed in HMLE cells as determined by qPCR). In two independent HEK293 clones with the 5′ splice site deleted, we observed that the level of detained intron 1 was increased, as was the level of circDOCK1(2–27) (Figure 7B). This result is consistent with the role of ESRP1 being simply to detain intron 1 while transcription proceeds to exon 27, creating the possibility of backsplicing of exon 27 to exon 2.

We next considered how ESRP1 binding detains intron 1. The ESRP1 binding sites are located at a distance (>15 kb) from the 3′ splice site (Figure 4A), but a possibility is that the extensive region of ESRP1 binding in intron 1 might act as a local sink for snRNPs or other essential splicing factors, thereby depleting the local concentration at the splice sites. Another possibility is that ESRP1 might competitively bind to a key spliceosome component to block its productive interactions during splicing. To gain insight into these possibilities, we performed a BioID analysis with tagged ESRP1 and searched the list of proximal proteins for evidence of such interactions with spliceosome components or other splicing factors. The list of high-confidence proximal interactors was analysed using g:Profiler, revealing strong enrichment for gene ontology molecular function (GO MF) term RNA binding (GO:0003723; *P*_adj_ = 5.17 × 10^−78^), though this included both cellular components (GO CC) of the cytoplasmic ribonucleoprotein granule (GO:0036464; *P*_adj_ = 4.43 × 10^−40^) and significant but lower enrichment of nuclear components including the spliceosomal complex (GO:0005681; *P*_adj_ = 2.87 × 10^−12^). KEGG and REACTOME pathway analysis both recovered spliceosome/mRNA splicing as top enriched categories (KEGG:03040; *P*_adj_ = 2.66 × 10^−7^; REAC:R-HSA-72163; *P*_adj_ = 1.33 × 10^−17^), though this was dominated by Heterogeneous nuclear ribonucleoproteins (HNRNPs) rather than core spliceosome components, with a few exceptions. Interpretation of the ESRP1 BioID data in the context of the humancellmap.org (a large project that aims to systematically use BioID data to reveal subcellular organization (31)), revealed the closest similarity in prey recovery profiles with HNRNPA1 (Jaccard distance 0.733; 52 common partners/195 total partners across the two baits). The prey recovery profile similarity to HNRNPA1 was higher than for spliceosome component DHX8 (Jaccard 0.958; 11 common partners/261; scatterplots in Supplementary Figure S7), further confirming these observations. In agreement with this observation, the most specifically enriched proximal interactions with ESRP1 – compared to all baits in the humancellmap.org (31) – were with alternative splicing regulators, rather than core spliceosome components (Figure 7C, Supplementary Table S2). Though it is not possible to exclude artifacts associated with tagging ESRP1 with the BioID enzyme, together, these data suggest that the function of ESPR1 may not be to disrupt the core spliceosome function directly, but rather to modify the RNP landscape of intron 1 in a way that disfavours communication of exon 2 with exon 1.

We next considered how the coupling of exon 27 to exon 2 might be achieved. The two principal mechanisms described to date for promoting backsplicing are base pairing between inverted Alu repeats in the introns flanking the back-spliced exons (37,38), potentially aided by binding of the splicing factor SFPQ (39), and looping mediated by a dimeric RNA binding protein (4). Although ESRP1 is not known to be dimeric, it is conceivable that individual qRRM domains (of which there are three) can bind to separated GGU motifs to promote looping. However, only two very small peaks of ESRP1 binding within exon 28 were detected in the HITS-CLIP analysis and these peaks were less than 1% of the size of the intron 1 peak. Because this does not seem to indicate a strong propensity for ESRP1-mediated looping of intron 27 to intron 1, we searched for inverted Alu repeats that may cause such looping. Using the NCBI BLASTn tool to align the intron 1 sequence to the intron 27 sequence revealed multiple examples of inverted Alu repeats (Figure 7D). Thus the backsplicing of exon 27 to exon 2 is likely to be augmented by base pairing between intron 1 and intron 27 (Figures 7E and Supplementary Figure S8). The fact that ENCODE eCLIP data (40) indicate binding of SFPQ throughout both intron 1 and intron 27 of DOCK1 in HepG2 cells is consistent with this mechanism. The cooperation of multiple RNA-binding proteins and splicing factors with inverted intronic repeats to regulate circRNA formation has been previously shown to occur in control of production of a circRNA from the Drosophila laccase2 gene (41), further supporting the concept of combined roles of RNA binding proteins and hairpin formation in regulating the formation of some circRNAs.

## Discussion

EMT confers motility on cells to allow tissue remodelling during embryogenesis, but can be recapitulated in part (called epithelial plasticity) by cancer cells to promote tissue invasion and metastasis (42). EMT is a highly coordinated process with many contributing regulators and effectors that act on cytoskeletal components to reconfigure the cytoarchitecture and enable cell motility, with DOCK1 protein being part of this crucial regulatory network. We show here that alternative splicing of the DOCK1 transcript to generate the circDOCK1(2–27) circular RNA is highly regulated in EMT and contributes to the regulation of the migratory capacity of cancer cells.

ESRP1 has been shown to contribute to enforcing epithelial phenotype (43–45), but has not been previously linked to regulation of DOCK1. However, the regulation and functions we have ascribed to circDOCK1(2–27) align well with the known roles of both ESRP1 and DOCK1. The DOCK1 protein is a guanine exchange factor (GEF), which in conjunction with its binding partner ELMO1 activates the GTPase Rac1, leading to cytoskeletal rearrangements that promote cell membrane spreading and cell migration (34,46). ESRP1 has been shown to control alternative splicing of multiple genes during EMT, many of which contribute to alterations in the actin cytoskeleton and cell motility (43,47,48). The effects we see of ESRP1 on both DOCK1 and circDOCK1(2–27) expression contribute to effects on cell motility, and provide a further example of the coordinated, multicomponent control of the cytoskeleton and motility that is evident in EMT. Since these are crucial capacities of cells that contribute to cancer metastasis, it would be interesting to assess the ability of circDOCK1(2–27) to suppress carcinoma metastasis.

Our crystallography data confirm that the ESRP1 qRRM2 recognises GGU rather than UGG, which is consistent with the results of previous SELEX and HITS-CLIP studies (44,49), although those studies, like our HITS-CLIP study, tended to identify the GGU sequence as being within a slightly longer GU-rich context. The very high degree of sequence similarity between the different qRRM domains of ESRP1 suggests to us that the RNA contacts will be very similar for all domains, but structural studies of intact, or multi-domain regions of the ESRP1 protein may clarify whether the additional flanking bases contribute to binding, and also whether multiple domains can bind to multiple adjacent GGU motifs to enhance affinity and/or affect functional effects of the RNA-bound ESRP1.

We note that while we did not see any effect of ESRP2 on circDOCK1(2–27), it is possible that any effect of depletion of ESRP2 was masked by the more abundant ESRP1 in HMLE cells. Given the high degree of sequence similarity between ESRP1 and ESRP2, we expect they would have similar RNA-binding profiles and that ESRP2 could regulate circDOCK1(2–27) formation in cells that have more abundant ESRP2. We speculate that the proximity labelling of ESRP2 by the ESRP1 bait in the BioID experiment we performed in HEK293 cells may be due to the two family members binding to adjacent sites, such as the reiterated sites we observed by HITS-CLIP analysis in the DOCK1 intron 1.

Whereas most circRNAs are expressed at very low levels such that despite their long half-lives they are much less abundant than their cognate mRNAs, we found that the backsplicing to produce the circDOCK1(2–27) circRNA is unusually efficient in cells that express ESRP1. The circDOCK1(2–27) ranks as the second most abundant circRNA in HMLE cells (behind circHIPK3), and among the top 10 circRNAs in expression relative to that of the cognate Mrna (4). The mode and degree of regulation of circDOCK1(2–27) is also unusual for a circRNA in that it is strongly reciprocal to the expression of mRNA from the host gene. This suggests its function is antithetically related to that of the cognate mRNA and this indeed appears to be very much the case since we find evidence of two simultaneous mechanisms that oppose function of the host gene in epithelial cells. Firstly, the formation of the circRNA reduces DOCK1 protein expression from the host gene by diverting transcripts from mRNA production to circRNA production. The efficiency with which this occurs in epithelial cells is remarkable given the large separation in sequence distance between the two exons that are ligated to form the RNA circle, with exon 2 separated from exon 27 by 157 kb. Also remarkable is the large number of exons in circDOCK1(2–27) (all 26 of which we have found are retained in the circRNA); most circRNAs have fewer than 5 exons and 99% of circRNAs are comprised of fewer than 12 exons (50,51). Secondly, the circRNA per se suppresses cell migration, as evidenced by the effect of its ectopic expression in inhibiting migration of mesenchymal cells, which has been previously shown in MDA-MB-231 breast cancer cells (52) and we show here in both mesHMLE and MDA-MB-231 cells.

Deep sequencing has revealed that many genes can produce multiple circRNAs with different backspliced ligations resulting in different exons incorporated, and the DOCK1 gene is typical in this regard. A number of previous publications have reported effects of ‘circDOCK1’ in various cancers, however those reports relate to different circRNAs and none of the reports, apart from our previous report on circDOCK1 in breast cancer cells (52) are on circDOCK1(2–27), but instead describe circRNAs with different circBase identifiers that are much less abundant than circDOCK1(2–27) (which has the circBase ID hsa_circ_0020397). To avoid possible ambiguity or confusion regarding circRNA identity, we suggest that any report focusing on the properties or functions of a specific circRNA should include a definitive description of the exons involved in backsplice formation, as recently proposed (53). Moreover, since many detected circRNAs are only expressed at an exceedingly low level, we propose a quantitative measure of the circRNA abundance be reported, to support the likelihood it is present in cells at a level commensurate with the proposed function.

## Supplementary Material

gkad1138_Supplemental_Files

## Data Availability

HITS-CLIP RNA-seq data were deposited to the NCBI GEO repository under accession number GSE226538. The mass spectrometry data was deposited to ProteomeXchange through partner MassIVE (massive.ucsd.edu) and assigned identifiers PXD040537 and MSV000091390.

## References

[1] Vicens Q. , Westhof E. Biogenesis of circular RNAs. Cell. 2014; 159:13–14. 25259915 10.1016/j.cell.2014.09.005

[2] Xu C. , Zhang J. Mammalian circular RNAs result largely from splicing errors. Cell Rep. 2021; 36:109439. 34320353 10.1016/j.celrep.2021.109439 PMC8365531

[3] Ashwal-Fluss R. , Meyer M., Pamudurti N.R., Ivanov A., Bartok O., Hanan M., Evantal N., Memczak S., Rajewsky N., Kadener S. circRNA biogenesis competes with pre-mRNA splicing. Mol. Cell. 2014; 56:55–66. 25242144 10.1016/j.molcel.2014.08.019

[4] Conn S.J. , Pillman K.A., Toubia J., Conn V.M., Salmanidis M., Phillips C.A., Roslan S., Schreiber A.W., Gregory P.A., Goodall G.J. The RNA binding protein quaking regulates formation of circRNAs. Cell. 2015; 160:1125–1134. 25768908 10.1016/j.cell.2015.02.014

[5] Teplova M. , Hafner M., Teplov D., Essig K., Tuschl T., Patel D.J. Structure-function studies of STAR family quaking proteins bound to their in vivo RNA target sites. Genes Dev. 2013; 27:928–940. 23630077 10.1101/gad.216531.113 PMC3650229

[6] Tran H. , Gourrier N., Lemercier-Neuillet C., Dhaenens C.M., Vautrin A., Fernandez-Gomez F.J., Arandel L., Carpentier C., Obriot H., Eddarkaoui S. et al. Analysis of exonic regions involved in nuclear localization, splicing activity, and dimerization of muscleblind-like-1 isoforms. J. Biol. Chem. 2011; 286:16435–16446. 21454535 10.1074/jbc.M110.194928 PMC3091249

[7] Chen S. , Huang V., Xu X., Livingstone J., Soares F., Jeon J., Zeng Y., Hua J.T., Petricca J., Guo H. et al. Widespread and functional RNA circularization in localized prostate cancer. Cell. 2019; 176:831–843. 30735634 10.1016/j.cell.2019.01.025

[8] Pillman K.A. , Phillips C.A., Roslan S., Toubia J., Dredge B.K., Bert A.G., Lumb R., Neumann D.P., Li X., Conn S.J. et al. miR-200/375 control epithelial plasticity-associated alternative splicing by repressing the RNA -binding protein Quaking. EMBO J. 2018; 37:e99016. 29871889 10.15252/embj.201899016 PMC6028027

[9] Van Nostrand E.L. , Shishkin A.a., Pratt G.a., Nguyen T.B., Yeo G.W. Variation in single-nucleotide sensitivity of eCLIP derived from reverse transcription conditions. Methods. 2017; 126:29–37. 28790018 10.1016/j.ymeth.2017.08.002 PMC5582984

[10] Martin M. Cutadapt removes adapter sequences from high-throughput sequencing reads. EMBnet.journal. 2011; 17:10.

[11] Smith T. , Heger A., Sudbery I. UMI-tools: modeling sequencing errors in Unique Molecular Identifiers to improve quantification accuracy. Genome Res. 2017; 27:491–499. 28100584 10.1101/gr.209601.116 PMC5340976

[12] Dobin A. , Davis C.A., Schlesinger F., Drenkow J., Zaleski C., Jha S., Batut P., Chaisson M., Gingeras T.R. STAR: ultrafast universal RNA-seq aligner. Bioinformatics. 2013; 29:15–21. 23104886 10.1093/bioinformatics/bts635 PMC3530905

[13] Li H. , Handsaker B., Wysoker A., Fennell T., Ruan J., Homer N., Marth G., Abecasis G., Durbin R. The Sequence Alignment/Map format and SAMtools. Bioinformatics. 2009; 25:2078–2079. 19505943 10.1093/bioinformatics/btp352 PMC2723002

[14] Zhang Y. , Liu T., Meyer C.A., Eeckhoute J., Johnson D.S., Bernstein B.E., Nussbaum C., Myers R.M., Brown M., Li W. et al. Model-based analysis of ChIP-Seq (MACS). Genome Biol. 2008; 9:R137. 18798982 10.1186/gb-2008-9-9-r137 PMC2592715

[15] Robinson J.T. , Thorvaldsdóttir H., Winckler W., Guttman M., Lander E.S., Getz G., Mesirov J.P. Integrative genomics viewer. Nat. Biotechnol. 2011; 29:24–26. 21221095 10.1038/nbt.1754 PMC3346182

[16] Heinz S. , Benner C., Spann N., Bertolino E., Lin Y.C., Laslo P., Cheng J.X., Murre C., Singh H., Glass C.K. Simple combinations of lineage-determining transcription factors prime cis-regulatory elements required for macrophage and B cell identities. Mol. Cell. 2010; 38:576–589. 20513432 10.1016/j.molcel.2010.05.004 PMC2898526

[17] Minor W. , Cymborowski M., Otwinowski Z., Chruszcz M. HKL-3000: the integration of data reduction and structure solution - From diffraction images to an initial model in minutes. Acta Crystallogr. Sect. D Biol. Crystallogr. 2006; 62:859–866. 16855301 10.1107/S0907444906019949

[18] Kabsch W. research papers XDS research papers. Acta Crystallogr. Sect. D Biol. Crystallogr. 2010; 66:125–132. 20124692 10.1107/S0907444909047337 PMC2815665

[19] Potterton E. , Briggs P., Turkenburg M., Dodson E. A graphical user interface to the CCP4 program suite. Acta Crystallogr. Sect. D Biol. Crystallogr. 2003; 59:1131–1137. 12832755 10.1107/s0907444903008126

[20] McCoy A.J. , Grosse-Kunstleve R.W., Adams P.D., Winn M.D., Storoni L.C., Read R.J. Phaser crystallographic software. J. Appl. Crystallogr. 2007; 40:658–674. 19461840 10.1107/S0021889807021206 PMC2483472

[21] Murshudov G.N. , Skubák P., Lebedev A.a., Pannu N.S., Steiner R.a., Nicholls R.a., Winn M.D., Long F., Vagin A.a REFMAC5 for the refinement of macromolecular crystal structures. Acta Crystallogr. Sect. D Biol. Crystallogr. 2011; 67:355–367. 21460454 10.1107/S0907444911001314 PMC3069751

[22] Emsley P. , Cowtan K. Coot: model-building tools for molecular graphics. Acta Crystallogr. Sect. D Biol. Crystallogr. 2004; 60:2126–2132. 15572765 10.1107/S0907444904019158

[23] Afonine P.V. , Grosse-Kunstleve R.W., Echols N., Headd J.J., Moriarty N.W., Mustyakimov M., Terwilliger T.C., Urzhumtsev A., Zwart P.H., Adams P.D. Towards automated crystallographic structure refinement with phenix.refine. Acta Crystallogr. Sect. D Biol. Crystallogr. 2012; 68:352–367. 22505256 10.1107/S0907444912001308 PMC3322595

[24] Lambert J.P. , Tucholska M., Go C., Knight J.D.R., Gingras A.C. Proximity biotinylation and affinity purification are complementary approaches for the interactome mapping of chromatin-associated protein complexes. J. Proteomics. 2015; 118:81–94. 25281560 10.1016/j.jprot.2014.09.011 PMC4383713

[25] Liu G. , Knight J.D.R., Zhang J.P., Tsou C.C., Wang J., Lambert J.P., Larsen B., Tyers M., Raught B., Bandeira N. et al. Data independent acquisition analysis in ProHits 4.0. J. Proteomics. 2016; 149:64–68. 27132685 10.1016/j.jprot.2016.04.042 PMC5079801

[26] Perkins D.N. , Pappin D.J.C., Creasy D.M., Cottrell J.S. Probability-based protein identification by searching sequence databases using mass spectrometry data. Electrophoresis. 1999; 20:3551–3567. 10612281 10.1002/(SICI)1522-2683(19991201)20:18<3551::AID-ELPS3551>3.0.CO;2-2

[27] Eng J.K. , Jahan T.a., Hoopmann M.R. Comet: an open-source MS/MS sequence database search tool. Proteomics. 2013; 13:22–24. 23148064 10.1002/pmic.201200439

[28] Shteynberg D. , Deutsch E.W., Lam H., Eng J.K., Sun Z., Tasman N., Mendoza L., Moritz R.L., Aebersold R., Nesvizhskii A.I. iProphet: multi-level integrative analysis of shotgun proteomic data improves peptide and protein identification rates and error estimates. Mol. Cell. Proteomics. 2011; 10:M111.007690. 10.1074/mcp.M111.007690 PMC3237071 21876204

[29] Teo G. , Liu G., Zhang J., Nesvizhskii A.I., Gingras A.C., Choi H. SAINTexpress: improvements and additional features in Significance Analysis of INTeractome software. J. Proteomics. 2014; 100:37–43. 24513533 10.1016/j.jprot.2013.10.023 PMC4102138

[30] Knight J.D.R. , Liu G., Zhang J.P., Pasculescu A., Choi H., Gingras A.C. A web-tool for visualizing quantitative protein-protein interaction data. Proteomics. 2015; 15:1432–1436. 25422071 10.1002/pmic.201400429

[31] Go C.D. , Knight J.D.R., Rajasekharan A., Rathod B., Hesketh G.G., Abe K.T., Youn J.Y., Samavarchi-Tehrani P., Zhang H., Zhu L.Y. et al. A proximity-dependent biotinylation map of a human cell. Nature. 2021; 595:120–124. 34079125 10.1038/s41586-021-03592-2

[32] Raudvere U. , Kolberg L., Kuzmin I., Arak T., Adler P., Peterson H., Vilo J. G:profiler: a web server for functional enrichment analysis and conversions of gene lists (2019 update). Nucleic Acids Res. 2019; 47:W191–W198. 31066453 10.1093/nar/gkz369 PMC6602461

[33] Warzecha C.C. , Sato T.K., Nabet B., Hogenesch J.B., Carstens R.P. ESRP1 and ESRP2 are epithelial cell-type-specific regulators of FGFR2 splicing. Mol. Cell. 2009; 33:591–601. 19285943 10.1016/j.molcel.2009.01.025 PMC2702247

[34] Koubek E.J. , Santy L.C. Actin Up: an overview of the Rac GEF Dock1/Dock180 and its role in cytoskeleton rearrangement. Cells. 2022; 11:3565. 36428994 10.3390/cells11223565 PMC9688060

[35] Dominguez C. , Fisette J.F., Chabot B., Allain F.H.T. Structural basis of G-tract recognition and encaging by hnRNP F quasi-RRMs. Nat. Struct. Mol. Biol. 2010; 17:853–861. 20526337 10.1038/nsmb.1814

[36] Herzel L. , Ottoz D.S.M., Alpert T., Neugebauer K.M. Splicing and transcription touch base: co-transcriptional spliceosome assembly and function. Nat. Rev. Mol. Cell Biol. 2017; 18:637–650. 28792005 10.1038/nrm.2017.63 PMC5928008

[37] Jeck W. , Sorrentino J., Wang K., Slevin M. Circular RNAs are abundant, conserved, and associated with ALU repeats. RNA. 2013; 19:141–157. 23249747 10.1261/rna.035667.112 PMC3543092

[38] Zhang X.-O. , Wang H.-B., Zhang Y., Lu X., Chen L.-L., Yang L. Complementary sequence-mediated exon circularization. Cell. 2014; 159:134–147. 25242744 10.1016/j.cell.2014.09.001

[39] Stagsted L.V.W. , O’leary E.T., Ebbesen K.K., Hansen T.B. The rna-binding protein sfpq preserves long-intron splicing and regulates circrna biogenesis in mammals. eLife. 2021; 10:e63088. 33476259 10.7554/eLife.63088 PMC7819710

[40] Dunham I. , Kundaje A., Aldred S.F., Collins P.J., Davis C.A., Doyle F., Epstein C.B., Frietze S., Harrow J., Kaul R. et al. An integrated encyclopedia of DNA elements in the human genome. Nature. 2012; 489:57–74. 22955616 10.1038/nature11247 PMC3439153

[41] Kramer M.C. , Liang D., Tatomer D.C., Gold B., March Z.M., Cherry S., Wilusz J.E. Combinatorial control of Drosophila circular RNA expression by intronic repeats, hnRNPs, and SR proteins. Genes Dev. 2015; 29:2168–2182. 26450910 10.1101/gad.270421.115 PMC4617980

[42] Yang J. , Antin P., Berx G., Blanpain C., Brabletz T., Bronner M., Campbell K., Cano A., Casanova J., Christofori G. et al. Guidelines and definitions for research on epithelial–mesenchymal transition. Nat. Rev. Mol. Cell Biol. 2020; 20:341–352. 10.1038/s41580-020-0237-9 PMC7250738 32300252

[43] Warzecha C.C. , Jiang P., Amirikian K., Dittmar K.a., Lu H., Shen S., Guo W., Xing Y., Carstens R.P. An ESRP-regulated splicing programme is abrogated during the epithelial-mesenchymal transition. EMBO J. 2010; 29:3286–3300. 20711167 10.1038/emboj.2010.195 PMC2957203

[44] Dittmar K.a. , Jiang P., Park J.W., Amirikian K., Wan J., Shen S., Xing Y., Carstens R.P. Genome-wide determination of a broad ESRP-regulated posttranscriptional Network by high-throughput sequencing. Mol. Cell. Biol. 2012; 32:1468–1482. 22354987 10.1128/MCB.06536-11 PMC3318588

[45] Lee S.K. , Cieply B., Yang Y., Peart N., Glaser C., Chan P., Carstens R.P. Esrp1-regulated splicing of Arhgef11 isoforms is required for epithelial tight junction integrity. Cell Rep. 2018; 25:2417–2430. 30485810 10.1016/j.celrep.2018.10.097 PMC6371790

[46] Gumienny T.L. , Brugnera E., Tosello-Trampont A.C., Kinchen J.M., Haney L.B., Nishiwaki K., Walk S.F., Nemergut M.E., Macara I.G., Francis R. et al. CED-12/ELMO, a novel member of the CrkII/Dock180/Rac pathway, is required for phagocytosis and cell migration. Cell. 2001; 107:27–41. 11595183 10.1016/s0092-8674(01)00520-7

[47] Bebee T.W. , Park J.W., Sheridan K.I., Warzecha C.C., Cieply B.W., Rohacek A.M., Xing Y., Carstens R.P. The splicing regulators Esrp1 and Esrp2 direct an epithelial splicing program essential for mammalian development. eLife. 2015; 4:e08954. 26371508 10.7554/eLife.08954 PMC4566030

[48] Ishii H. , Saitoh M., Sakamoto K., Kondo T., Katoh R., Tanaka S., Motizuki M., Masuyama K., Miyazawa K. Epithelial splicing regulatory proteins 1 (ESRP1) and 2 (ESRP2) suppress cancer cell motility via different mechanisms. J. Biol. Chem. 2014; 289:27386–27399. 25143390 10.1074/jbc.M114.589432 PMC4183779

[49] Peart N.J. , Hwang J.Y., Quesnel-Vallières M., Sears M.J., Yang Y., Stoilov P., Barash Y., Park J.W., Lynch K.W., Carstens R.P. The global Protein-RNA interaction map of ESRP1 defines a post-transcriptional program that is essential for epithelial cell function. iScience. 2022; 25:105205. 36238894 10.1016/j.isci.2022.105205 PMC9550651

[50] Ragan C. , Goodall G.J., Shirokikh N.E., Preiss T. Insights into the biogenesis and potential functions of exonic circular RNA. Sci. Rep. 2019; 9:2048. 30765711 10.1038/s41598-018-37037-0 PMC6376117

[51] Xin R. , Gao Y., Gao Y., Wang R., Kadash-Edmondson K.E., Liu B., Wang Y., Lin L., Xing Y. isoCirc catalogs full-length circular RNA isoforms in human transcriptomes. Nat. Commun. 2021; 12:266. 33436621 10.1038/s41467-020-20459-8 PMC7803736

[52] Kurosaki M. , Terao M., Liu D., Zanetti A., Guarrera L., Bolis M., Gianni’ M., Paroni G., Goodall G.J., Garattini E A dock1 gene-derived circular rna is highly expressed in luminal mammary tumours and is involved in the epithelial differentiation, growth, and motility of breast cancer cells. Cancers (Basel). 2021; 13:5325. 34771489 10.3390/cancers13215325 PMC8582367

[53] Chen L. , Bindereif A., Bozzoni I., Chang H.Y., Matera A.G., Gorospe M., Hansen T.B., Kjems J., Ma X., Pek J.W. et al. A guide to naming eukaryotic circular RNAs. Nat. Cell Biol. 2023; 25:1–5. 36658223 10.1038/s41556-022-01066-9 PMC10114414

[54] Vo J.N. , Cieslik M., Zhang Y., Shukla S., Xiao L., Zhang Y., Wu Y.M., Dhanasekaran S.M., Engelke C.G., Cao X. et al. The landscape of circular RNA in cancer. Cell. 2019; 176:869–881. 30735636 10.1016/j.cell.2018.12.021 PMC6601354

