# Supplementary material for: ESRP1 controls biogenesis and function of a large abundant multiexon circRNA
Source: Nucleic Acids Res. 2023 Nov 28;52(3):1387–403. doi: 10.1093/nar/gkad1138 (PMC10853802; doi:10.1093/nar/gkad1138)
Supplement: gkad1138_Supplemental_Files [file gkad1138_supplemental_files.zip › Supplementary Figures.pdf]

SUPPLEMENTARY INFORMATION

Table S1. The correlation coefficients of mRNAs with circDOCK1(2,27), from all tumours in the miOncoCirc database,[43] related to Figure 2E.

Table S2. Results from SAINT (Significance Analysis of INTeractome) analysis of the BioID mass spec data.

Table S3 Summary of crystallography data collection and structural refinement statistics.

Table S4 Sequences of all oligonucleotides used in the study.

Figure S1 related to Figure 1 CircDOCK1(2,27) is epithelial-specific and regulated during EMT.
(A) Back-splice sequence of the circDOCK1(2,27) from Sanger sequencing of the product from circDOCK1(2,27) qPCR in Figure 1. Additionally, the qPCR Ct values for circDOCK1 and DOCK1 mRNA from HMLE and mesHMLE cells are provided.
(B) Intron and exon structure of the DOCK1 gene.
(C) Ct values for the qPCR measurements of DOCK1 mRNA and circDOCK1(2-27) in various breast cancer cell lines.
(D) ZEB1 mRNA in TGFβ-treated HMLE cells measured by qRT-PCR. Mean +/- SEM. n = 3 biological replicates, performed in technical triplicate.
(E) Tumor samples in the MiOncoCirc database (v0.1.release, Vo et al., 2019) were in-silico classified into epithelial or mesenchymal groups based on the parental gene expression of eight EMT marker genes (epithelial markers: CDH1, ESRP1, ESRP2 and CLDN7 and mesenchymal markers: CDH2, VIM, ZEB1 and ZEB2). Of the 876 samples in the parental gene expression matrix, 218 (25%) showed an expression pattern consistent with that for an epithelial state, 165 (19%) showed a mesenchymal pattern, while 493 (56%) showed a lack of concordance between markers and were subsequently excluded from further analyses.

Figure S2 related to Figure 2 ESRP1 regulates the circDOCK1(2,27) level.
(A,B) Changes in ESRP1 and ESRP2 level after siRNA knockdown of ESRP1 or ESRP2. Mean +/- SEM. n = 3 biological replicates, performed in technical triplicate. *** $P < 0.001$, ns: not significant. ordinary one-way ANOVA analysis. Additionally, the qPCR Ct values for ESRP1 and ESRP2 mRNA are provided.

(C) Time courses of circDOCK1(2,27) and ESRP1 expression in HMLE and MCF10A cells treated with TGFβ. Error bars show SEM of triplicate qPCR assays.

Figure S3 related to Figure 3.

(A) Western blots of DOCK1, ESRP1, E-cadherin and tubulin levels in epithelial and mesenchymal cell lines, with quantitation of DOCK1 in the right panel.
(B) Schematic diagram of qPCR primers detecting circDOCK1 and linear RNA product from the construct expressing circDOCK1(2-27) in transfected cells. The forward convergent PCR primer is designed to target the intron-exon junction, enabling the detection of linear unspliced RNA derived from the construct. The divergent primers are specifically designed to detect circDOCK1(2-27) expression.

(C) RT-qPCR validation of circDOCK1 and the liner RNA product derived from the circDOCK1 expressing construct in stable mesHMLE cells. Mean +/- SEM. n = 3 biological replicates, performed in technical triplicate. Additionally, the qPCR Ct values for circDOCK1, the linear RNA product, and the housekeeping gene GAPDH are provided.
(D) Representative images from the Transwell migration assays.

Figure S4 related to Figure 4 ESRP1 Regulates Formation of circDOCK1 via Binding Sites in Intron 1.
(A) The sequence of the region of DOCK1 intron 1 encompassing broad secondary peaks of ESRP1 binding identified by HITS-CLIP.

Figure S5 related to Figure 5 Crystal structure of the ESRP1 RRM2-RNA complex.
(A) Sequence alignments of the ESRP1 and hnRNA F RRM domains, which suggests that ESRP1 has quasi-RRM domains like hnRNP F.
(B) Sequence alignments of the ESRP1 qRRM domains showing structural elements and key residues of domain 2 that form the two G clamps.

Figure S6 related to Figure 6 ESRP1 detains DOCK1 Intron 1 to facilitate circDOCK1 back splicing.
(A) Sanger sequencing of the region encompassing deletion of the ESRP1-binding region in DOCK1 intron 1.
(B) ESRP1 levels, determined by qPCR, are unchanged on deletion of the ESRP1 binding sites in DOCK1 intron 1.

Figure S7 related to Figure 7
Scatterplots showing the overlaps between the preys detected with ESRP1 and those detected for HNRNPA1 or DHX8 in the humancellmap.org. Blue colour coding indicate the confidence level (high-confidence preys are those with a Bayesian FDR of $\leq$1%) with at least one of the baits compared. The green colour coding indicates members of the HNRNP protein family. Figure generated from ProHitz-viz.org[12].

Figure S8 related to Figure 7 Potential base pairing of Alu repeats in introns 1 and 27.
Sequence alignments of Alu elements in intron 1 and intron 27 that can potentially base pair to promote backsplicing.

A

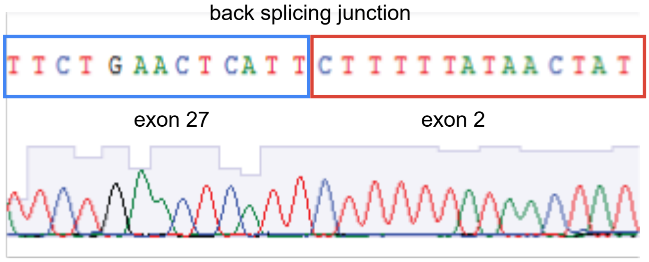

	circDOCK1		DOCK1 mRNA		GAPDH	
	HMLE	mesHMLE	HMLE	mesHMLE	HMLE	mesHMLE
Replicate 01	22	26.5	21.4	21.6	11	12.6
Replicate 02	23.3	27.4	23.2	22.2	12.7	12.8
Replicate 03	22.6	26.3	22.2	21.1	12.6	12.2

B

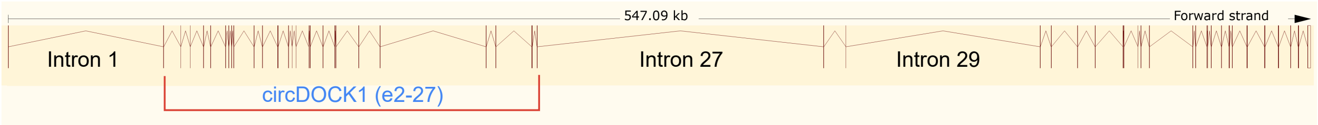

C

	epithelial							
	MCF-7	T47-D	MM-361	ZR-75-1	MM-415	MM-134		
GAPDH	13.4	14.7	12.6	12.4	13.3	14.4		
circDOCK1	19.2	18.9	18.2	19.9	19.7	18.9		
DOCK1 mRNA	22.7	25	21.8	24	21.6	24.1		
	mesenchymal							
	BT549	HS578T	MM-231	MM-436	MM-157	CAL51	CAL120	SUM159PT
GAPDH	12.3	12.5	12.7	12.3	13.9	12.5	12.8	12.1
circDOCK1	26.1	26.5	25.7	26.3	24.4	26.5	27.5	26.1
DOCK1 mRNA	20.8	20.8	21	21.7	20.5	20.4	21.4	21

D

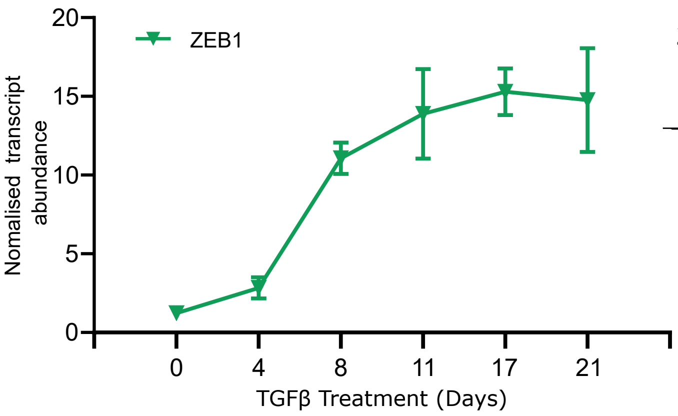

E

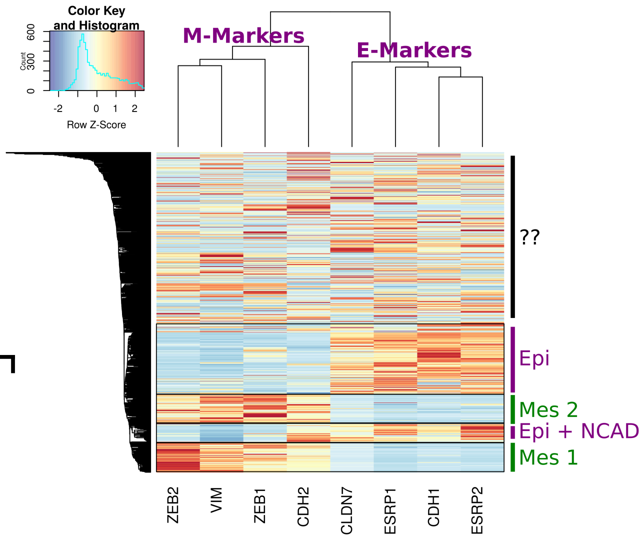

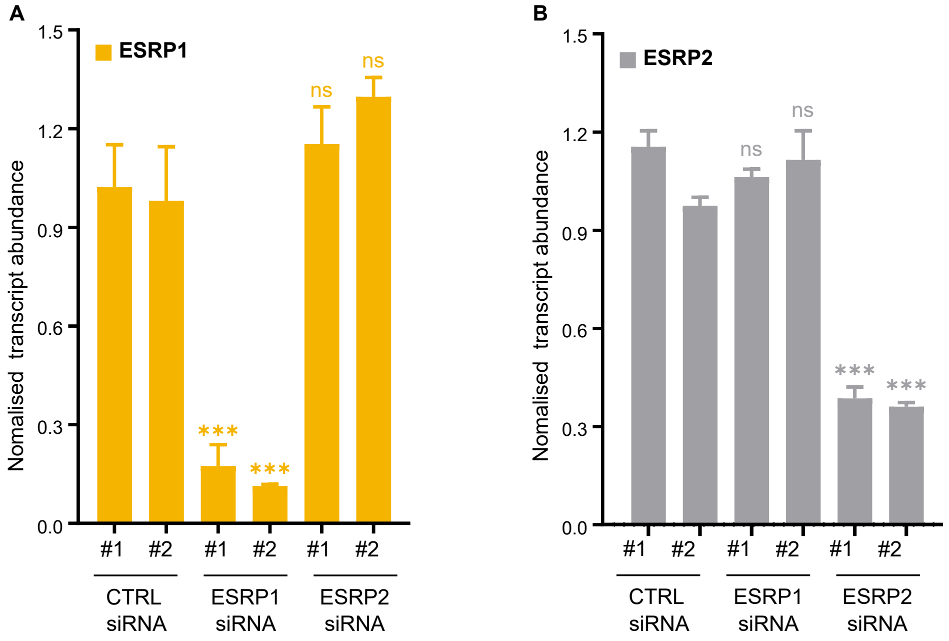

	ESRP1 qPCR Ct Value					
	CTRL #1	CTRL #2	ESRP1 #1	ESRP1 #2	ESRP2 #1	ESRP2 #2
Replicate01	17.9	17.7	21	22.2	17.8	18.1
Replicate02	17.9	18	20.5	21.7	18.2	17.6
Replicate03	19.1	19.3	23.2	22.6	18.9	18.5

	ESRP2 qPCR Ct Value					
	CTRL #1	CTRL #2	ESRP1 #1	ESRP1 #2	ESRP2 #1	ESRP2 #2
Replicate01	19.2	19.5	19.5	20	21.2	21.6
Replicate02	19.4	19.6	19.7	18.8	21.7	21.5
Replicate03	19.7	20	19.9	20.2	21.9	22.3

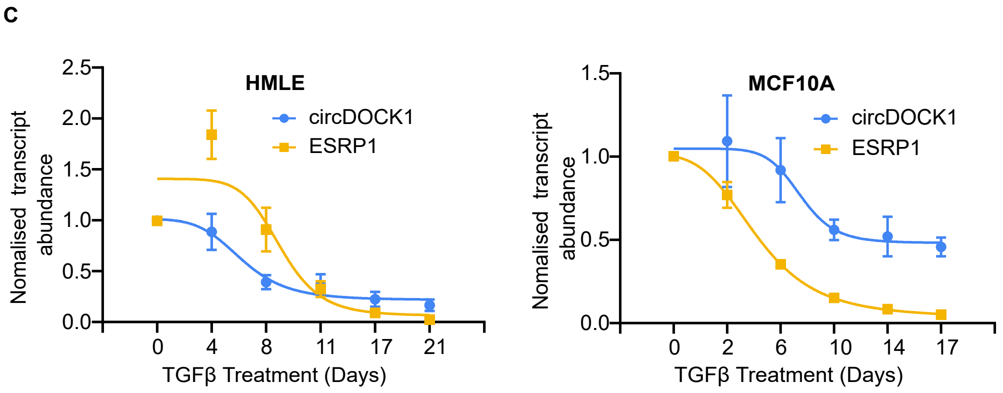

A

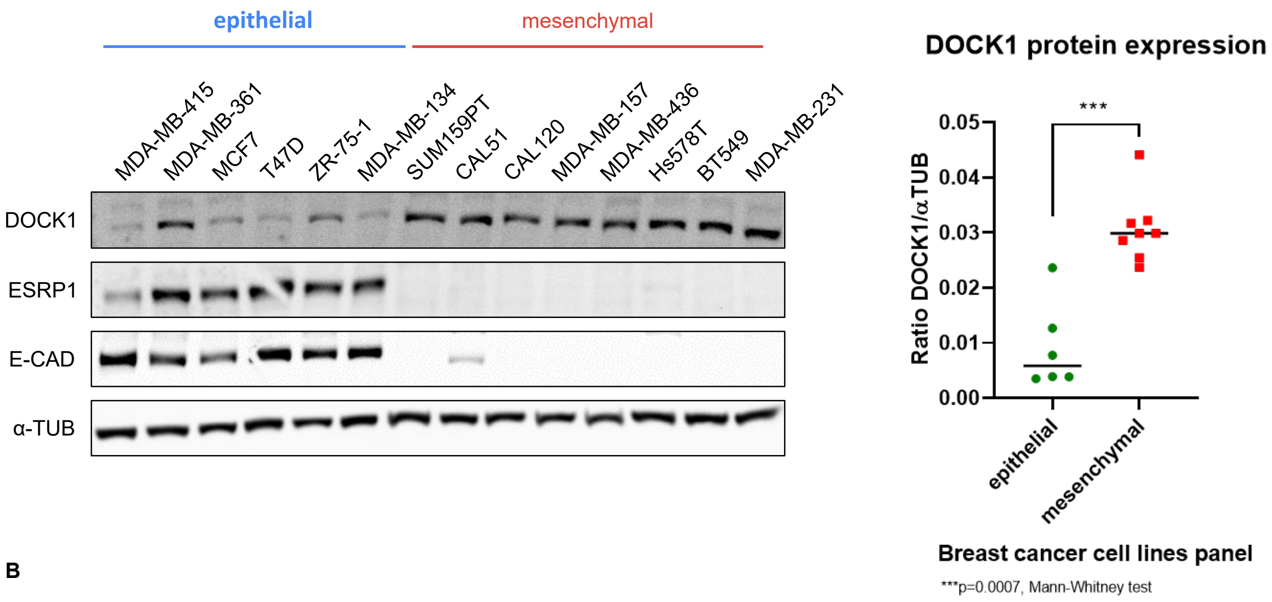

B

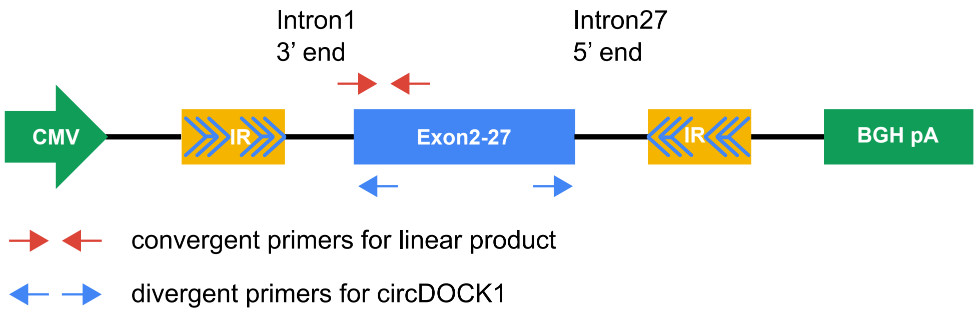

C

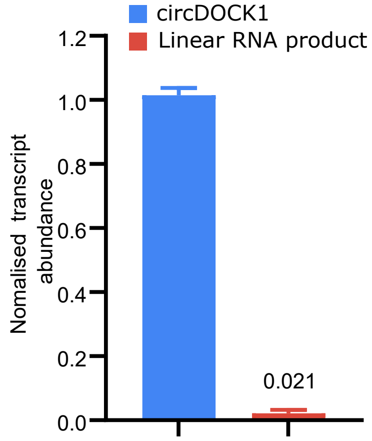

	qPCR Ct Value		
	GAPDH	circDOCK1	Linear RNA product
Replicate1	10.7	17	25.8
Replicate2	13.4	20.7	26.9
Replicate3	12.5	19.1	25.8

D

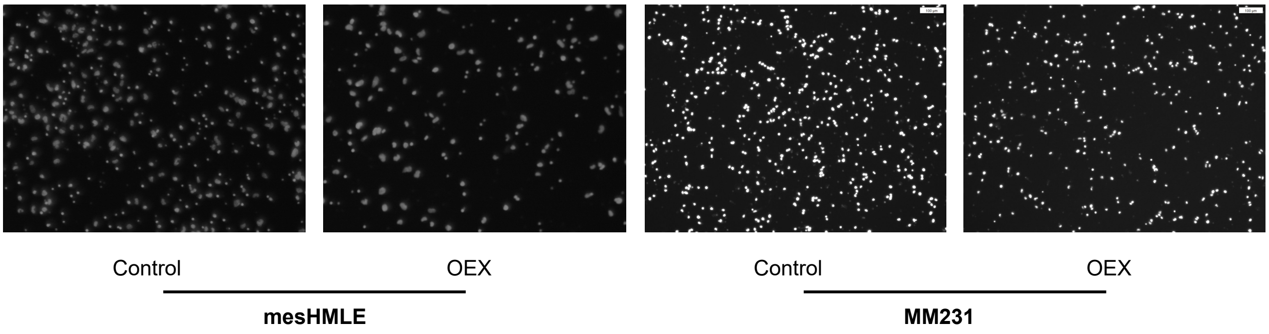

A

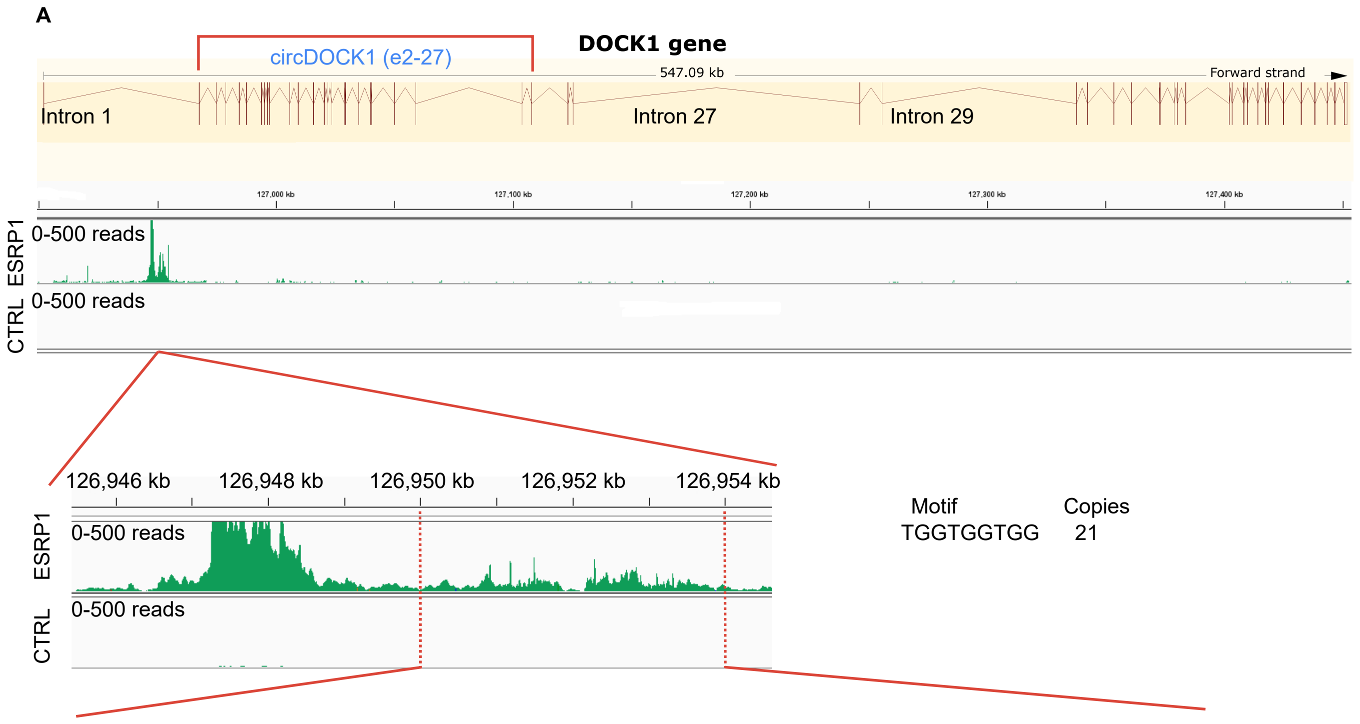


```
>hg38_dna range=chr10:126950001-126954000 5'pad=0 3'pad=0 strand=+ repeatMasking=none
CCCACCTTCTAGACCTGCGTCTTTGAGGAGCCATCTAACATCCTCTCTAAGCCTCATTTTTTTTTTCCATTTGTGAAATGGGAATGAGTAGTAATCCCTC
CCTTGTAAAGTTTTTGTGATAATGAAATTAGATAAATTAGGTAGTCCACGAGAAGCAGTCAGTGGCTGTTCCATGGTAAGGGGCTAATAGCTCTCATTAC
AGCTTTTCCTAGTTGTGTTATTTGGAGAGCAGGTTCCTAATTCGAGTTCGCCACAGCTTATCTGTGTCATGTCTTGTCTCCTCTTTGGGTGGCAGTTCTT
ACCTGGGATCTTGGGTGGTAAATATTGGTTTGTTGGTCTGGATTGACTGGATGATTGGAGGTCTGGTCTGTGCTTGCCTGCAGGGTCAGCTTAGCCTAGC
TGTGACATGTAAAGGCTCTCTCTCCTTTAGACTGGGGTTCCATTCATGGGATTAATGGAATTGTTATTTCATCTTGTCTCAAGGGCTTTCTGGAATAAGG
GCAATGGGGCCTTTGACAGTGCCCAGAATTGTCACTGTCCCTTTGAAGCAAAGGGAAATCGGTTGCAGAGAGAGGGACTCTGCAGCCTTTGTTGTGGACG
CAGCAGTACAGTTACTCTTTGTTTGGGATGTGCTGGGAGTGCTGTTCTCAGAGCAAGGCCACATGATGCCTGGAGGTGGGGCGGCAGCATGCCCTCCTTC
CTCCTGCTCTTTGCCTCAGAGTCTTTGTGTGCAGAAATGGGGTTTTTGTTAGCCCCACTTCATAGGGTTAATATGGGATGATGTGAGGAAATGCGTCAAG
TGTTCGTTGGAGTTTCTGGTCTTTCCCAGGGCAGAATGAAGTTTAACTCTACCATGATGATGGTGGTACTGGTGATAGCGGTGGTGCTGGTGATGTTGGT
GATGTTATTGTTGATGATGGTGGTTGGTAGTATTGCTGTTGGCGGTGATGGTGGTGGTGGTAACATTGCTGGCGGTGATTGTGTTGGTAGTATTGATGTT
GGTGGGGGTGGTGAAGGTGGTAGTATTGTTGGTAGTAGTAGTATTGGTGATGGTGGTGGTAGTATTGTTTTTGGTAGTGTTTTTAGTATTGATGATAGTG
GTGGTAGTGTTGTTGGTAGTATTGTTGGTAGTGTTAGCATGGTTGTTGGTGGTAGTATTACTGGTAGTAGTGTTGGTGATAGTGGTGATGGTGGTAGTAT
TGGTAGTGGTGGTGGTAATATTGGTGATGGTGGTAGTATTGTTGGTAATATTAGTGATAGTGGTGGTGGTAGTATTGTTGGTAGTATTGGTAGTGTTAGT
AGTGGTAGTATTGTTGGTAGTATTGTTGGTGATAGTGGTGATGGTAGTATTGTTGATAGTATTGGTAGTGGTGGTGGTAGTATTGGTGATGGTAGTAGTA
TTGCTAATATTAGTGATAGTGGTGGTGGTAGTATTGTTGGTAGTGTTGGTAGTATTGGTAGTAGTGGTAGTATTCGTAGTATTGTTGGTGATAGTGGTGA
TGGTGGTAATGTTGTTTTTGTTGGTATTATTGCTGTTGGTAGTATTGTTGGTGATGTGTTGGTGGTGATTGTGGTGGTGGTAGTATTGTTGGTAGTGTTT
TTGGTAGTGTTGGTGGTGGTATTGTTGGTGGTAGTATTGTTGTTTGTAGTATTGGTGACAATGGTAGTAGTAGTAAGTATTGTTGTTGGTATTACTAGTA
TTGAGGCAGAAGAATAGAGCCTGGAGGCAGAAGACCTAAGGACTTCCTAGAACTAAATCAAATGGAAACACGCTGTGACAGGAAATAATCTCTTCATTTA
CATTGGGTGTACACTGAGTAAATGACTATGTAACTTTACTTCAGCCTCTTCATTTTTTTTTTTTTTTGAGATGGAGTTTCACTATTGTCACCCAGGCTGGA
GTGCAGTGGTGCGATCTCGGCTCACTGCAACCTCCGCCTCCTGGGTTCAAGCGATTTTCCTGCCTCGGACTCCTGAGTAGCTGGGACTACAGATGTGTAC
CACCAGGCCCAGCTAATTTTTGTATTCTTAGTAGAGATGGGATTTCACCATGTTGGCCAGGATGGTATCAATTTCTTGACCTTGTGATCCGCCCACCTCG
GCCTCCCAGAGGGGTGGGATTACAGGCATGAGCTACCATGCCTGGCCAGCCTCTTCGTTTATATAGGGTGTACACCAAGTAACCAATGGTGGTGGTAGTA
TTGTTGGTGATACGGTAGTATTGTTATTGTTGGTAGTGTTGTAGGTGATGGTGATGGTAATAGCATTGTTATTGTTCATAGTATTGTAGGTGATGGTGGT
GGTGGCAGTAGTATTGTTATTGTTGGTTGTATTGTTGGTGATGGTGGTGGTAATATTGTTACTGTTGGTAGTATTGTTGGTAGTGTTGGTGATGTGGCGG
TAGTATTGTTGGTAGTGTTGGTGATGTTATTCTTGGTAGTATTGTTGGTGATGGTGGTGGTGGTAGTATTGGTGGTAGTATTGTTGGTGATGTATTATTG
GTAGTGTTGGTGTTGTTATTCTTGGTAGTACGGTTGGTGATGGTGGTGGTAGTATTGGTGGTAGTGTTGTTGGTGATGTAGTATTGTTGGTACTGTTGGT
GATGTTATTCTTGGTAGTATTGTTGGTGATGTAGTATTGTTGGTAGTGTTGGTGATGTTGTTATTGGTAGTATTGTTGTTGTTGGTAGTATTGTTGGTGA
TGTGGTAGTATTGTTGGTAGTGTTGGTGATGTTGTTATTGGTAGTATTGGTGGTAGTATTGTTGGTGATGTGGTAGTATTGTTGGTAGTGTTGGTGATGT
TATTCTTGGTAGTATTGTTGGTGATGGTGGTGGTGGTAGTGTTGGTAGTATTGTTGGTGATGTGGTAGTATTGTTATTGTTGGTAGTATTGTAGGTGATG
GTGGTGGTGATAGTATTGTTATTGTTGGTAGTATTGTTGGTGATGGTAGTGGTAGTATTTTTTGTTGGTGGTATTGTTGGTAGTATAGTTAGTGATGTAG
TGTTGGTGGTGGTAGTATTGTTGGTGCTGCTGGTGGTGGTAGTATTTTGGTAGTGGTGGTAGTATTTTTGGTAGTGGTGGTAGTATTTTGGTGATGGTT
GTGAAGTATTGGTGATGGTGGTGGTGGAAGTATTGGTAGTAATTTTCGTAGTGTTGGTGGTGGTGGTATTGTTATTGGCAGCGATGGCAGTGGAGGTGGT
AGTGGTGGTAGTACTGTTGGTAGTATTTTTAGTAGTGTTGGTAGTGGTGGTATTGGTGATGGTGGTGGTGGTAGTGTTGTGGTGTGGTGTGGTGGTAGTG
GTGATGGCAGTGGAGGTGGTAGTGGTGGTAGTATTGTCAGTGATGGTGGTTGTGGTAGTATTGTTGGTAGTATTTTTCATAGTGTTGGTAGTGGTGGTAT
TGGTGATGGTGGTGGTGGTAGTATTGTTATCGGTGGTGATGGCGGTGGAGGTGGTGATGGCAGTGGAGGTGGTAGTGGTAGCAATATTATTGATAGCAGT
TTTGGTAATGTTGGTGGTGGTAGTACTGCTGATGGTGGTGGTGGTGGTAGTATTGTTAATCAGTGGTGATGGCAGGGGAGGTGCTGGTGTTAGTACACTG
TCATCAGTGGAGCTCCCATTTTTCAGCTGAATGCCTTCGTGGCATAGGCCTCCTGGGAATGAGTGTGGGTAGAACGTCCCTCATTTGTGGTTGAGATTTT
TCCCTGGAGCTGTGTTGGTGGGGGAAGCCTTGTTCCACGTGTGTTGGGGTTAGAGGGTGTGTGGCTGCTCAGTGGTCAGCTTTGATGTCACCTGAAACAT
GTTACAGGTGAGTCAGGTGGAGCATTGGGGAACTGCAGGGAGAGGAGTGTTTTCTGATGTCAGAGCCGATGGAGCTTTAAAAGCTAAAAATGAAACAAAC
AGAAAATGTGGAGAGATGTTCAGTAGTTTGCTCGGTACATTTTTAAAACTTCCGAGTGGTCCTGTGTGTTGGGTGTCATGGTGGACACACTGTCGTGGGC
```

A

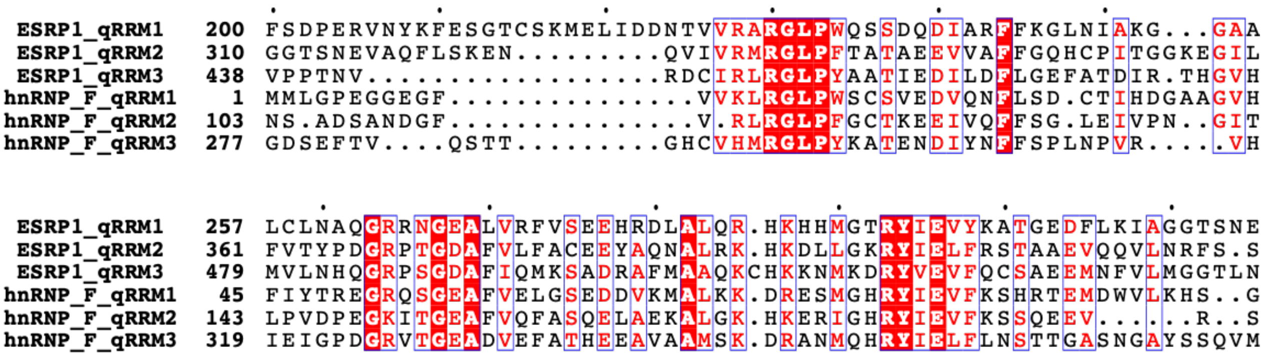

B

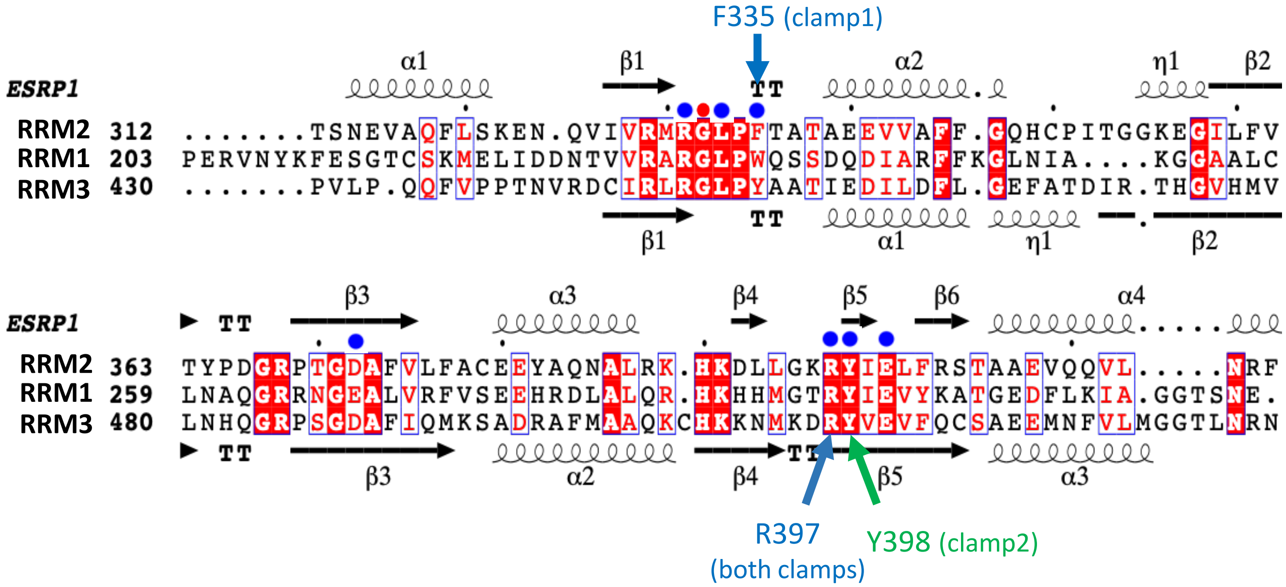

Figure S5

(A) Alignment of the ESRP1 and hnRNPF qRRM domain sequences.

(B) Sequence alignment of the three qRRM domains with residues involved in RNA binding are indicated by blue or red circles. The red circle indicates the interaction is with the main chain.

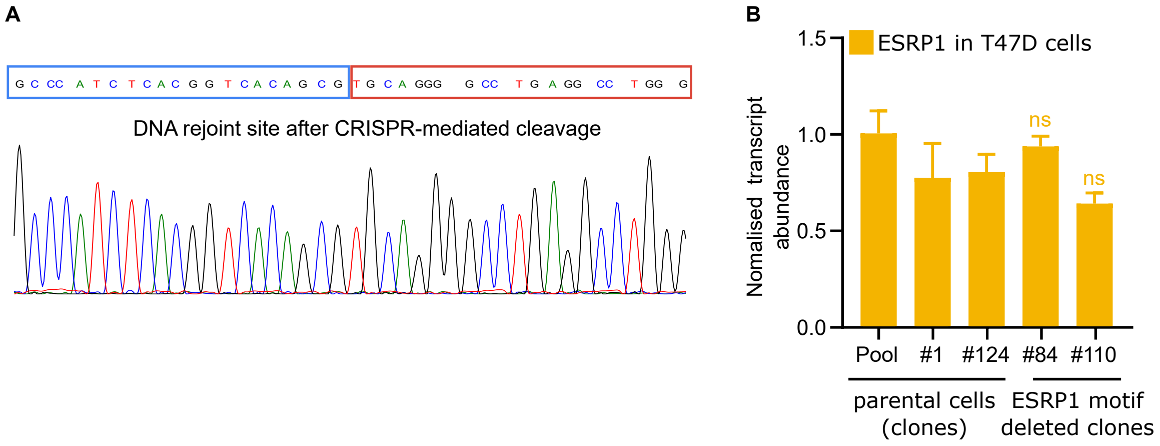

Figure S6 related to Figure 6 ESRP1 detains DOCK1 Intron 1 to facilitate circDOCK1 back splicing.

(A) Sanger sequencing of the region encompassing deletion of the ESRP1-binding region in DOCK1
intron 1.

(B) ESRP1 levels, determined by qPCR, are unchanged on deletion of the ESRP1 binding sites in DOCK1 intron 1.

Figure S7

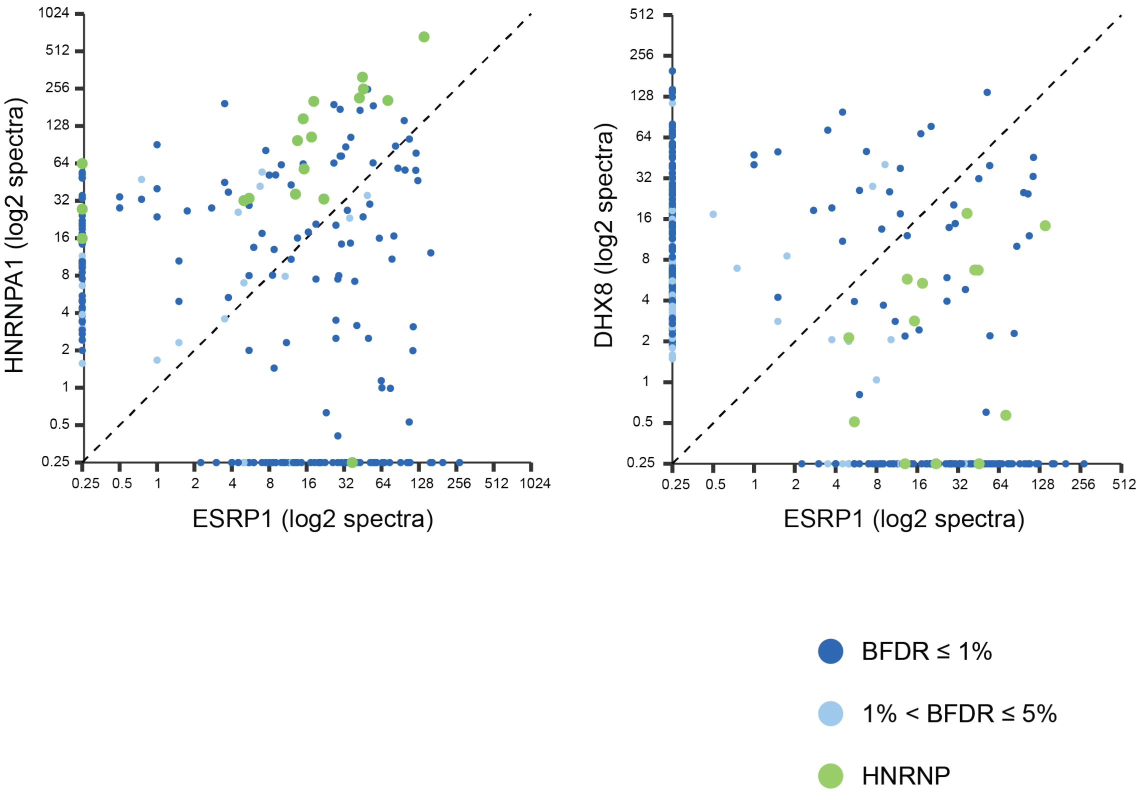

Figure S7 related to Figure 7
Scatterplots showing the overlaps between the preys detected with ESRP1 and those detected for HNRNPA1 or DHX8 in the humancellmap.org. Blue colour coding indicate the confidence level (high-confidence preys are those with a Bayesian FDR of ≤1% with at least one of the baits compared. The green colour coding indicates members of the HNRNP protein family. Figure generated from ProHitz-viz.org12.

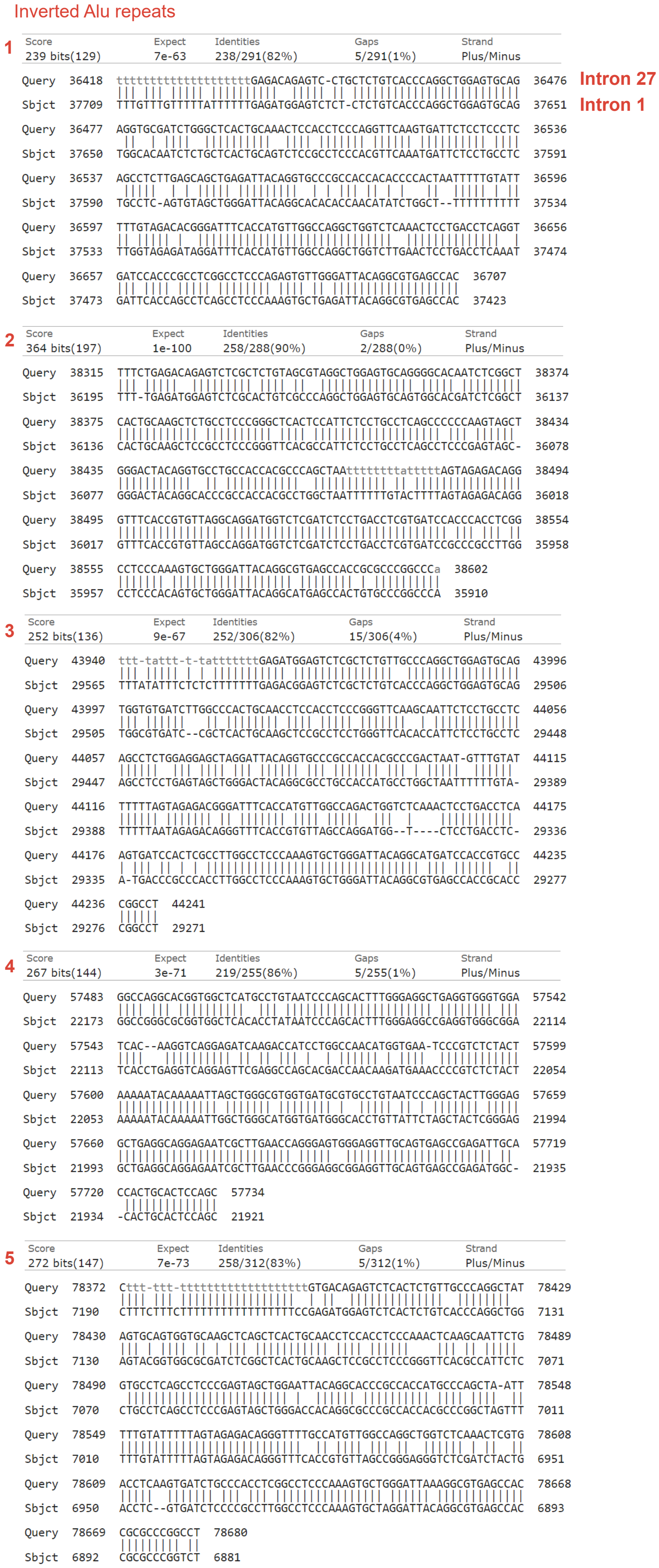

Figure S8 related to Figure 7 Potential base pairing of Alu repeats in introns 1 and 27. Sequence alignments of Alu elements in intron 1 and intron 27 that can potentially base pair to promote backsplicing.
