# Supplementary material for: ESRP1 controls biogenesis and function of a large abundant multiexon circRNA
Source: Nucleic Acids Res. 2023 Nov 28;52(3):1387–403. doi: 10.1093/nar/gkad1138 (PMC10853802; doi:10.1093/nar/gkad1138)
Supplement: gkad1138_Supplemental_Files [file gkad1138_supplemental_files.zip › Table S3.pdf]

Table S3. Data collection and refinement statistics.

	qRRM2-12mer-RNA (7VKI)
**Data collection**	
Space group	$P3_12_1$
Cell dimensions	
a, b, *c* (Å)	86.12, 86.12, 43.28
α, β γ, (°)	90, 90, 120
Wavelength(Å)	0.94989
Resolution (Å)	30.00-1.65 (1.71-1.65)
R_{merge}	0.145
$I / \sigma I$	17.42 (6.31)
Completeness (%)	99.8(98.6)
Redundancy	11.8
**Refinement**	
Resolution (Å)	28.25-1.65
No. reflections	21026
$R_{\mathbf{work}} / R_{free}$	0.1944/0.2217
No. atoms	1081
Protein	887
RNA	66
Water	128
Average *B*-factor (Å)	14.38
**R.m.s. deviations**	
Bond lengths (Å)	0.006
Bond angles (°)	0.797
**Ramachandran plot**	
Favored/allowed (%)	100.0/0.0
